# The Sole DNA Ligase in *Entamoeba histolytica* Is a High-Fidelity DNA Ligase Involved in DNA Damage Repair

**DOI:** 10.3389/fcimb.2018.00214

**Published:** 2018-07-12

**Authors:** Elisa Azuara-Liceaga, Abigail Betanzos, Cesar S. Cardona-Felix, Elizabeth J. Castañeda-Ortiz, Helios Cárdenas, Rosa E. Cárdenas-Guerra, Guillermo Pastor-Palacios, Guillermina García-Rivera, David Hernández-Álvarez, Carlos H. Trasviña-Arenas, Corina Diaz-Quezada, Esther Orozco, Luis G. Brieba

**Affiliations:** ^1^Posgrado en Ciencias Genómicas, Universidad Autónoma de la Ciudad de México, Mexico City, Mexico; ^2^Consejo Nacional de Ciencia y Tecnología, Mexico City, Mexico; ^3^Departamento de Infectómica y Patogénesis Molecular, Centro de Investigación y de Estudios Avanzados del Instituto Politécnico Nacional, Mexico City, Mexico; ^4^Laboratorio Nacional de Genómica para la Biodiversidad, Centro de Investigación y de Estudios Avanzados, Irapuato, Mexico

**Keywords:** EhDNAligI, protozoan, DNA insults, ligation, repairing, 8-oxoG adduct, NER and BER pathways

## Abstract

The protozoan parasite *Entamoeba histolytica* is exposed to reactive oxygen and nitric oxide species that have the potential to damage its genome. *E. histolytica* harbors enzymes involved in DNA repair pathways like Base and Nucleotide Excision Repair. The majority of DNA repairs pathways converge in their final step in which a DNA ligase seals the DNA nicks. In contrast to other eukaryotes, the genome of *E. histolytica* encodes only one DNA ligase (EhDNAligI), suggesting that this ligase is involved in both DNA replication and DNA repair. Therefore, the aim of this work was to characterize EhDNAligI, its ligation fidelity and its ability to ligate opposite DNA mismatches and oxidative DNA lesions, and to study its expression changes and localization during and after recovery from UV and H_2_O_2_ treatment. We found that EhDNAligI is a high-fidelity DNA ligase on canonical substrates and is able to discriminate erroneous base-pairing opposite DNA lesions. EhDNAligI expression decreases after DNA damage induced by UV and H_2_O_2_ treatments, but it was upregulated during recovery time. Upon oxidative DNA damage, EhDNAligI relocates into the nucleus where it co-localizes with EhPCNA and the 8-oxoG adduct. The appearance and disappearance of 8-oxoG during and after both treatments suggest that DNA damaged was efficiently repaired because the mainly NER and BER components are expressed in this parasite and some of them were modulated after DNA insults. All these data disclose the relevance of EhDNAligI as a specialized and unique ligase in *E. histolytica* that may be involved in DNA repair of the 8-oxoG lesions.

## Introduction

*Entamoeba histolytica*, the protozoan parasite responsible for human amoebiasis, must cope with reactive oxygen (ROS) and nitric oxide (NOS) species derived from human immune cells, during colonic tissue invasion and metronidazole drug treatment (Vicente et al., 2009; Wilson et al., 2012). ROS and NOS species generate DNA lesions such as 8-oxoguanine (8-oxoG), thymine glycol (Tg) and pyrimidine dimers (Demple and Harrison, 1994). 8-oxoG is the most abundant DNA lesion formed during oxidative stress. During UV and H_2_O_2_ treatments, oxygen radicals generate 8-oxoG (Dianov et al., 1998). To avoid deleterious mutations this lesion is repaired primarily via the base excision repair (BER) pathway, which is present in two modalities: short and long-path (Lindahl, 1993). 8-oxoG is also recognized and processed by the nucleotide excision repair (NER) pathway, suggesting a cross talk between NER and BER to repair non-bulky oxidative DNA lesions (Menoni et al., 2012; Parlanti et al., 2012). DNA replication and repair are multi-enzymatic reactions that require the orchestrated participation of several enzymes, each with a specific task. For instance, in eukaryotic cells the interactions between DNA ligase I with PCNA and DNA polymerase β are critical for Okazaki fragment maturation and DNA repair, respectively (Dianov et al., 1998). DNA ligase I is recruited to UV-damage sites only in proliferating cells and it is implicated in BER and NER pathways (Moser et al., 2007). In humans the deficiency in DNA ligase I induces sunlight sensitivity, suggesting a role of this enzyme in DNA double-strand break repair (Bhat et al., 2006). DNA ligase III is involved in NER following UV-damage in quiescent cells, single-strand, and double stranded break repair; whereas DNA ligase IV is involved in DNA double-strand breaks repaired by the NHEJ pathway (Tomkinson et al., 2006).

In contrast to vertebrates, the protozoan parasite *E. histolytica* contains only one DNA ligase dubbed EhDNAligI (Cardona-Felix et al., 2010). EhDNAligI is similar to DNA ligase I from higher eukaryotes, however, its N-terminal is approximately 150 amino acids shorter than its human counterpart. The enzymatic activity of EhDNAligI is stimulated by the DNA polymerase processivity factor PCNA (Buguliskis et al., 2007; Cardona-Felix et al., 2010, 2011). The lack of other ATP or NAD^+^-dependent DNA ligases in the genome of *E. histolytica* suggests that EhDNAligI could participate in repairing diverse DNA lesions and sealing Okazaki fragments. Additionally, DNA sequencing of the genome of *E. histolytica* reveals that this parasite contains genes of the BER and NER pathways, including glycosylases, an apurinic endonuclease, excision repair proteins and helicases (Loftus et al., 2005; Clark et al., 2007; López-Camarillo et al., 2009). Some NER proteins of *E. histolytica* are overexpressed in response to UV irradiation (Weber et al., 2009).

In *E. histolytica* few proteins involved in DNA metabolism such as EhDNAligI, EhPCNA, EhMutY, specialized DNA polymerases, Myb transcription factors, and EhRad51 have been biochemically characterized (López-Casamichana et al., 2008; Cardona-Felix et al., 2010, 2011; Meneses et al., 2010; Pastor-Palacios et al., 2010, 2012; Trasviña-Arenas et al., 2016). Because EhDNAligI is the sole ligase in *E. histolytica*, we addressed its role during DNA damage induced by UV and H_2_O_2_ treatments. We tested its fidelity and ligation capabilities in sealing different DNA lesions *in vitro* and analyzed its changes in expression and localization upon DNA damage.

## Materials and methods

### Nick-sealing ligation reactions

Recombinant EhDNAligI (rEhDNAligI) was heterologously expressed, purified and deanylated as previously described (Cardona-Felix et al., 2010). T4 DNA ligase was purchased from New England Biolabs and deadenylated using molar excess of pyrophosphate. Ligation reactions were performed in 50 mM Tris pH 7.5, 1 mM ATP, 10 mM MgCl_2_, and 5 mM DTT. Double stranded DNA nicked substrates were annealed accordingly to the figure legends. Nick-sealing reactions were carried out at 37°C using 50 fmol of EhDNAligI and 250 fmol of nicked ^32^P labeled substrates. Reactions were stopped by adding equal volumes of stop buffer (95% formamide, 0.01% bromophenol blue, 1 mM EDTA). Samples were run on a 15% polyacrylamide, 8 M urea denaturing gel and analyzed on a phosphorimager (Storm; GE Healthcare) to quantify the results. All experiments were performed by triplicate.

### Electrophoretic mobility shifts assays

To determine the DNA binding specificity of rEhDNAligI to different lesions in comparison to a canonical substrate, we prepared three different substrates for DNA mobility shift assays. Oligonucleotides were mixed at equimolar amounts in an annealing buffer (20 mM Tris pH 7.5 and 150 mM NaCl), heated at 95°C for 5 min and slowly cooled to room temperature. Binding experiments were carried out with 300 fmol of DNA substrate and 26, 52, and 104 fmol of rEhDNAligI. Complex formation was analyzed by EMSA.

### Steady-state kinetics

Michaelis-Menten steady-state kinetics for ATP consumption was measured using 500 fmol of each of the three substrates. In all cases, the 5′ substrate was phosphorylated with γ-^32^P ATP. ATP concentrations varied from 3.125 to 800 nM. Ligation reactions were separated by electrophoresis and quantified on the phosphorimager. The Km and Kcat values were determined by fitting to the Michaelis–Menten equation as previously described (Buguliskis et al., 2007; Cardona-Felix et al., 2010).

### Survey of BER and NER gene encoding proteins in *E. histolytica* genome

Sequences annotated as NER and BER proteins were obtained from *E. histolytica* database (http://amoebadb.org/). For each component of the amoeba NER and BER proteins we searched for its putative orthologous in *Homo sapiens, Saccharomyces cerevisiae*, and other species using blast (http://blast.ncbi.nlm.nih.gov) against NCBI RefSeq protein data base restricting to the corresponding organism or the whole data base. The Percentage Identity (PID) was calculated using amino acids sequences from NER and BER proteins from *E. histolytica, H. sapiens, S. cerevisiae* and the organism corresponding to the best hit, taking gaps into account using the following equation: PID = (identical positions/length of the alignment) × 100. Oligonucleotides of the selected NER and BER genes (Table S1) were designed according to the DNA sequence obtained from NCBI RefSeq protein data base.

### *E. histolytica* cultures

Trophozoites of HM1:IMSS strain were axenically cultured in TYI-S-33 medium supplemented with 15% of bovine serum at previously described (Diamond et al., 1978) and used in logarithmic growth phase for all experiments.

### Trophozoites treatments

Trophozoites from 24 h cultures were subjected to two different types of stress: UV light irradiation and exposure to H_2_O_2_. For the first treatment, 5 × 10^5^ trophozoites were irradiated with 254 nm UV-C light at 150 J/m^2^ using a UV Stratalinker 1800 device (Stratagene) at previously described (López-Casamichana et al., 2008). For H_2_O_2_ treatment, 8 × 10^5^ trophozoites were exposed to 2.5 mM of H_2_O_2_ diluted in serum-depleted medium to avoid its protective effect during oxidative stress derived from peroxide, at 37°C for 1 h, as previously described (Shahi et al., 2016). After treatments, cells were allowed to recover in fresh TYI-S-33 complete medium at 37°C during 1, 3, 6, and 24 h. Trophozoites were chilled for 10 min and parasites were harvested at 1,500 rpm. Cell viability was determined using a TC20 Automated Cell Counter (Bio-Rad) using Trypan blue dye exclusion assays (Bio-Rad) and trophozoites with 80% viability were used for further experiments.

### RT-PCR and qRT-PCR assays

Total RNA from trophozoites either from the untreated control or immediately after treatment with UV or H_2_O_2_ was isolated using TRIzol (Life Technologies), following the manufacturer's protocol. cDNA was synthesized using 500 ng of total RNA with an oligo(dT)15 (Promega). The PCR reactions contained 1 μl of cDNA and 0.4 μmol of each specific oligonucleotide combination (Table S1). RT-PCR products were resolved in 2% agarose gels, stained with ethidium bromide and visualized with a FLA-5100 Imaging System (FUJIFILM). qRT-PCR was performed using the QuantiFast SYBR Green RT-PCR kit (Qiagen) and 100 ng of the total RNA, according to the manufacturer's instructions. Relative changes in gene expression were calculated using *40s rps2* as an internal gene calculated by ΔΔCT method according to the Guide to Performing Relative Quantitation of Gene Expression Using Real-Time Quantitative PCR from Applied Biosystems. All experiments were performed in triplicate and repeated in independent experiments by duplicate.

### Production of polyclonal antibodies against EhDNAligI and EhPCNA

rEhDNAligI and rEhPCNA recombinant proteins were heterologously expressed and purified as previously described (Cardona-Felix et al., 2010, 2011) and subsequently used as antigens to immunize New Zealand male rabbits. Before immunization, the pre-immune serum from rabbits was obtained; rabbits were subcutaneously inoculated with an initial dose of 130 μg rEhDNAligI or EhPCNA diluted in Freund's complete adjuvant (Sigma-Aldrich) and 4 more booster injections (130 μg each) at 21-day intervals. After 1 week of the last immunization, rabbits were bleed and polyclonal antisera were obtained. Reactivity of the generated antibodies was tested by Western blot assays, using rEhDNAligI and rEhPCNA recombinant proteins (Figure S1).

### Western blot assays

Lysates from trophozoites either untreated control, immediately after treatment with UV or H_2_O_2_ or during different recovery times (1, 3, and 6 h) were obtained using trichloroacetic acid (TCA; Sigma-Aldrich) method. Proteins were separated by 12% denaturing polyacrylamide gel electrophoresis (SDS-PAGE) and transferred onto nitrocellulose membranes (Millipore). Membranes were blocked with 5% nonfat dried milk and incubated for 18 h at 4°C with rabbit α-EhDNAligI (1:1,000) or α-γH2AX (1:7,000 dilution; Cell Signaling) antibodies or rabbit pre-immune serum (1:1,000) or mouse α-actin (1:1,500; antibody; Manning-Cela et al., 1994) followed by incubation for 2 h at room temperature with peroxidase-conjugated goat α-rabbit (1:5,000 dilution; Santa Cruz Biotechnology) or α-mouse (1:10,000 dilution; Invitrogen) secondary antibodies. Membranes were developed using an enhanced chemiluminescence kit (Luminata Forte Western HRP Substrate, Millipore), in the Image Lab 5.2.1 System (Bio-Rad). All experiments were performed in triplicate and repeated in independent experiments by duplicate.

### Immunofluorescence and determination of the 8-oxoguanine (8-oxoG) formation by confocal microscopy

The 8-oxoG formation was analyzed using a direct fluorescence-based binding assay with avidin-FITC (fluorescent dye, Sigma-Aldrich). Avidin binds with high affinity to 8-oxoG as Struthers et al. described (Struthers et al., 1998). Trophozoites untreated or treated with UV or H_2_O_2_ or recovered at different times (1, 3, 6, and 24 h) were fixed with absolute ethanol for 20 min at −20°C, washed with PBS pH 6.8 and blocked with 50 mM NH_4_Cl for 30 min at 37°C, followed by incubation of 1% BSA for 30 min at 37°C. Cells were incubated with 5 μg/mL FITC-conjugated avidin (1:50) for 1 h at room temperature in darkness. Then, samples were incubated with rabbit α-EhDNAligI (1:200) antibody overnight at 4°C. Next day, cells were washed with PBS pH 6.8 and incubated with α-rabbit TRITC-labeled secondary antibody (1:100; Jackson Immuno Research). Nucleic acids were counterstained with 2.5 μg/mL DAPI (4′,6′-diamidino-2-phenylindole; Zymed) for 5 min. Preparations were washed with PBS and fluorescence was preserved using antifade reagent Vectashield (Vector Lab). For some experiments, α-EhDNAligI (1:200) and α-EhPCNA (1:200) antibodies were Alexafluor 647 and 555 labeled, respectively using the Antibodies Labeling Kit (Molecular Probes), following the manufacturer's instructions. Light optical sections were obtained through a Nikon inverted microscope attached to a laser confocal scanning system (Leica Microsystems) and analyzed by Confocal Assistant Software Image. The integrated density fluorescence of the nucleus was quantified using the ImageJ software and it was normalized regarding the fluorescence of the whole cell in 5 different fields of three Z-stack sections (0.5 μm) of independent experiments. Nuclear co-localization was quantified in 1 μm *z*-stacks confocal images to obtain the Pearson Coefficient using the Just Another Co-localization Plugin (JACoP) in the Image J 1.48i software. Each point represented an average of 10–20 cells and values are given as means and standard error.

### Statistical analyses

All data shown are displayed as mean with standard error in triplicate and repeated in independent experiments by duplicate. GraphPad Prism 6.0e software was used for student *t*-test by two-tailed analyses.

### Ethics statement

The Institutional Animal Care and Use Committee (Cinvestav IACUC/ethics committee) reviewed and approved the animal care and use of rabbits and employed to produce antibodies (Protocol Number 0313-06, CICUAL 001). All steps were taken to ameliorate the welfare and to avoid the suffering of the animals. Rabbits were housed under controlled conditions of humidity, temperature, and light (12 h light/12 h dark cycles). Food and water were available *ad libitum*. Animals were monitored pre and post-inoculation. All procedures were conducted by trained personnel under the supervision of veterinarians and all invasive clinical procedures were performed while animals were anesthetized and when it was required animals were humanely euthanized. The ethics committee verified that our institute fulfills the NOM-062-ZOO-1999 regarding the Technical Specifications for Production, Care, and Use of Laboratory Animals given by the General Direction of Animal Health of the Minister of Agriculture of Mexico (SAGARPA-Mexico). The technical specifications approved by SAGARPA-Mexico fulfill of the international regulations/guidelines for the use and care of animals used in laboratory and were verified and approved by Cinvestav IACUC/ethics committee (Verification Approval Number: BOO.02.03.02.01.908).

## Results

### rEhDNAligI is a high-fidelity DNA ligase

**Figure 1:**
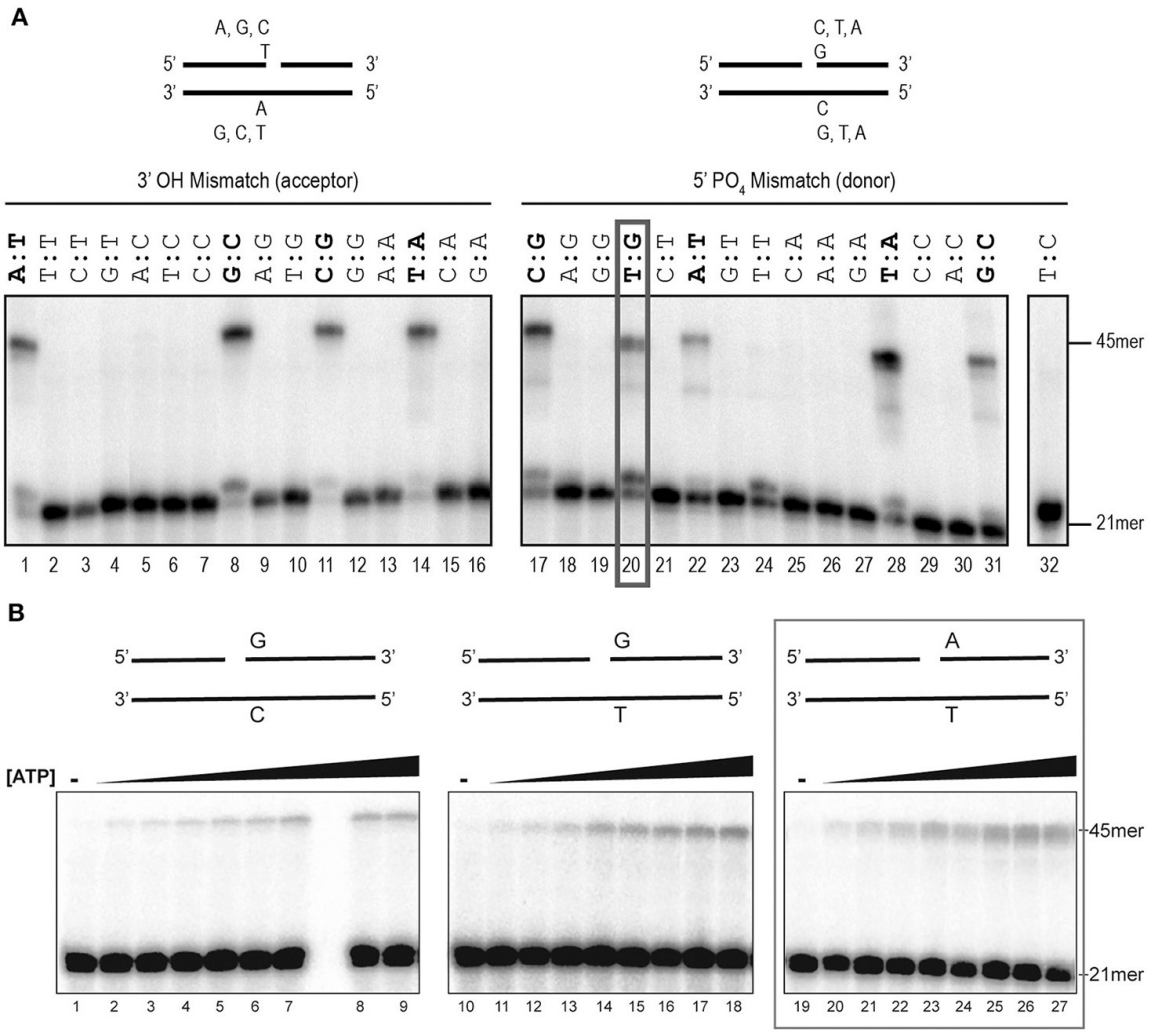
Assessment of rEhDNAligI fidelity on nicked double stranded DNA mismatches. **(A)** Schematic representation of the different substrates used to evaluate DNA ligation fidelity. The mismatches were evaluated at the 3′-OH (acceptor) and 5′-PO_4_ (donor) using a combination of 16 different oligonucleotides. Each substrate varies only one base at a time at the 3′-OH (acceptor, lanes 1–16) or 5′-PO_4_ (donor, lanes 17–32). Of the 16 combinations at each end, 4 of them do not have any mismatch and are marked in red, while 24 substrates have an error in base pairing. The red ox shows the only mismatched substrate ligated by EhDNAligI. **(B)** Steady-state kinetics of EhDNAligI on the G:T mismatch at different concentrations of ATP. Lanes 1–9 show EhDNAligI activity at different ATP concentrations in comparison to canonical G:C substrate, lanes 10–18 monitored ligase activity with a mismatched G:T substrate, lanes 19–27 indicate activity with the canonical A:T substrate. The 21mer substrate and the 45mer ligation products are marked with arrows.

To initiate the biochemical characterization of EhDNAligI, we purified the rEhDNAligI recombinant protein and performed a nick-sealing *in vitro* assay. Nick-sealing fidelity in DNA ligases relates to the extent to which the enzyme can ligate substrates containing mismatched bases on either side of the nick. Thus, we tested the 12 possible DNA mismatches at the 3′ and 5′ sides of a double-stranded DNA nick (Figure 1A). We found that rEhDNAligI was able to discriminate almost all the non-canonical Watson-Crick base pairs either at the 3′-OH or 5′-PO_4_ of the nicked DNA. rEhDNAligI only efficiently ligated the T:G mismatch at the 5′- PO_4_ strand (Figure 1A, lane 20). DNA ligation kinetics for a G:T mismatch in comparison to ligation employing G:C and T:A canonical pairs indicated that the mismatch and the canonical pairs are ligated with similar catalytic efficiencies that differ by no more than two-fold in their Km. This data indicates the among the 12 possible Watson-Crick errors, EhDNAligI only efficiently recognizes the T:G mismatch as a substrate for ligation (Figure 1B; Table 1). G:T mismatches occurs by deamination of 5-methylcytosine to thymine, the lack of substrate discrimination by EhDNAlig I is also observed in *Thermus thermophilus* DNA ligase (Luo et al., 1996).

**Table 1:** Steady-state kinetics constants for canonical bases and G:T mismatch.

**Kinetics constants**	**G:C**	**G:T**	**A:T**
Kcat (min^−1^)	2.4	2.039	2.208
Km (nM)	64	92	30

### rEhDNAligI is able to discriminate across from DNA lesions

**Figure 2:**
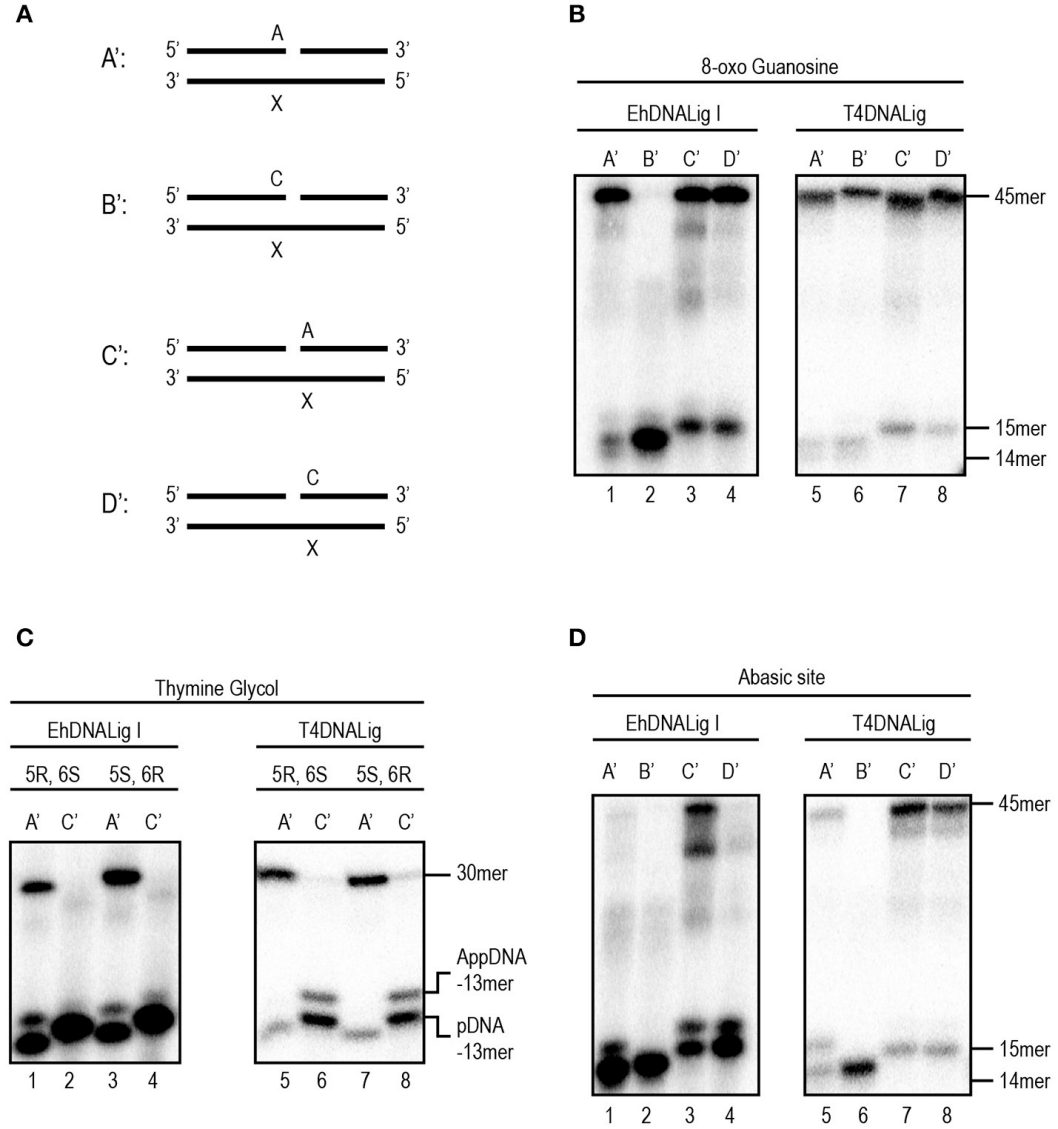
EhDNALigI activity on substrates with oxidative stress damage. **(A)** Schematic representation of the assayed substrate to measure DNA ligation at damaged substrates. The “X” indicates the location of three oxidative damages: 8-oxoG, thymine glycol and abasic site. In all cases, the damage is located at the template strand, while the complementary strand may have an adenine or cytosine, located at the 3′-OH (acceptor) (A' and B') or 5′-PO_4_ (donor) (C' and D'). **(B)** Ligation reaction with 8-oxoG template. Enzymatic activity against damaged substrates with different combinations as shown in **(A)** using EhDNAligI (lanes 1–4) or T4 DNA ligase (lanes 5–8). **(C)** Ligation reaction with thymine glycol template. Enzymatic activity against damaged substrates with different combinations as shown in **(A)** using EhDNAligI (lanes 1–4) or T4 DNA ligase (lanes 5–8). **(D)** Ligation reaction with abasic site. Enzymatic activity against damaged substrates with different combinations as shown in **(A)** using EhDNAligI (lanes 1–4) or T4 DNA ligase (lanes 5–8). pDNA indicates the phosphorylated substrate DNA, while AppDNA indicates adenylated substrate by a DNA ligase. The substrate and the ligation product are labeled with arrows.

DNA is susceptible to damage from endogenous or exogenous sources. Oxidative damage, including species such as singlet oxygen atoms, hydroxyl radicals, and superoxide radicals, alters the coding specificity of the nitrogenous bases. Thus, we tested four possible substrates (Figure 2A) with different base modifications related to different oxidative stress and compared the ability of rEhDNAligI and T4 DNA ligase to use those substrates. As 8-oxoG is the most common oxidative lesion generated by ROS, we tested the ligation efficiency of different substrates that contained 8-oxoG either at the 3′-OH or 5′-PO_4_ (Figure 2B). rEhDNAligI was able to ligate a dATP:8-oxoG pair but not a dCTP:8-oxoG pair at the 3′-OH (Figure 2B lanes 1, 2), in contrast, T4 DNA ligase was able to use as a substrate dCTP and dATP paired to an 8-oxoG lesion (Figure 2B lanes 5, 6). This substrate discrimination is similar to the one present in human DNA ligase I (Hashimoto et al., 2004). 8-oxoG lesion, at the 5′-PO_4_ of the lesion, is efficiently ligated when paired with dATP or dCTP by EhDNAligI and T4 DNA ligase (Figure 2B lanes 3, 4, 7, 8). 5,6-dihydroxy-5,6-dihydrothymine (thymine glycol), is another important DNA lesion induced by oxidation and ionizing radiation. In this work, we used two thymine glycol isomers, the 5R-6S and the 56-6R isomers and we tested whether rEhDNAligI discriminates between them. rEhDNAligI and T4 DNA ligase efficiently ligated a thymine glycol 5R-6S isomer and a 56-6R isomer at the 3′-OH position (Figure 2C lanes 1, 3 and 5, 7, respectively). However, rEhDNAligI and T4 DNA ligase were inefficient to perform a nick sealing reaction when thymine glycol is located at the 5′-PO_4_ (Figure 2C lanes 2, 4 and 6, 8, respectively). Finally, an abasic site occurs when the base is excised from the chain by spontaneous hydrolysis of the N-glycosylic bond. rEhDNAligI was unable to ligate an abasic site 3′-OH of the nick (Figure 2D lanes 1, 2); however, T4 DNA ligase moderately catalyzed the reaction, when dATP was present opposite to the lesion (Figure 2D lane 5). rEhDNAligI efficiently ligated an abasic site 5′-PO_4_ to the lesion only when dATP is complementary to the lesion but not dCTP (Figure 2D lanes 3, 4). In contrast, T4 DNA ligase efficiently ligated an abasic site 5′-PO_4_ to the nick opposite a purine and a pyrimidine (Figure 2D lanes 7, 8).

**Figure 3:**
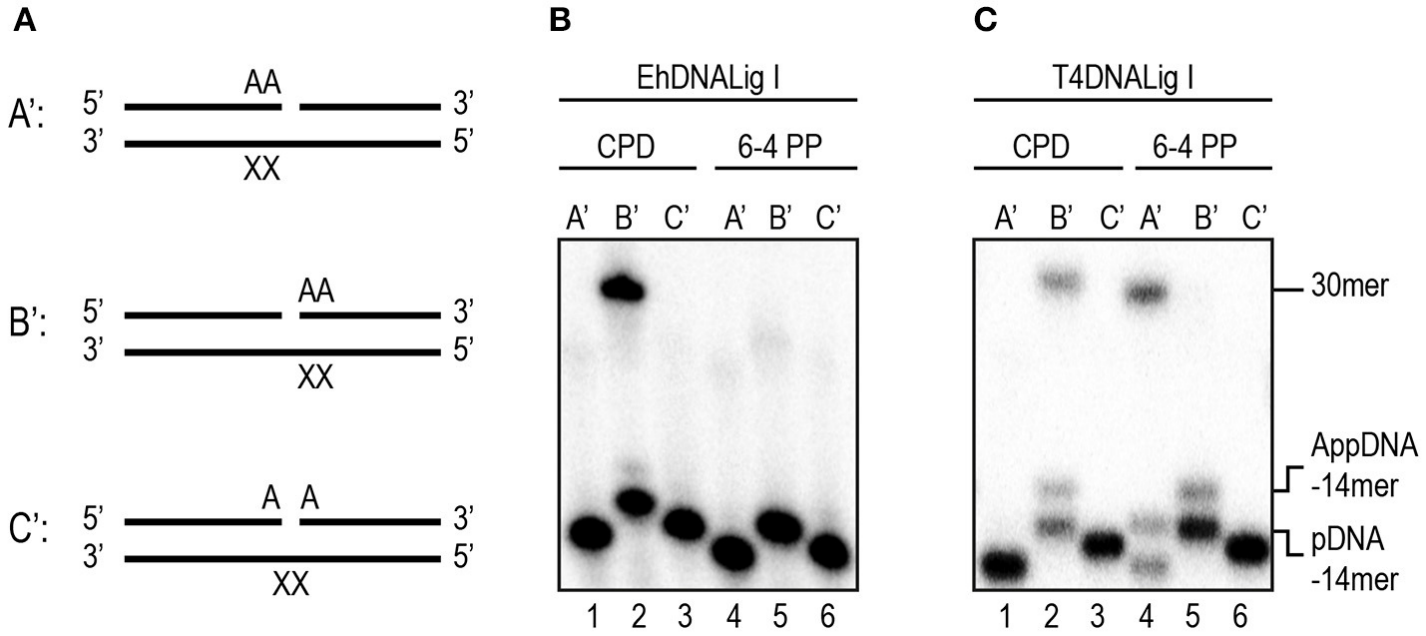
EhDNALigI nick-sealing activity of substrates with UV radiation damage. **(A)** Schematic representation of the substrates used to evaluate DNA ligation of UV-damaged DNA templates. The thymine dimers (CPD or 6-4PP) are represented as “XX.” In the three cases, the damage is located at the template strand, while the complementary strand contains two adenines at 3′-OH end in A', 5′-PO_4_ in B' or the nick (one at the 3′-OH and one at the 5′-PO_4_) as in C'. **(B)** Nick-sealing reactions with EhDNALigI, with substrates containing CPD (lanes 1–3) or 6-4PP (lanes 4–6). **(C)** As in **(B)**, but using T4 DNA ligase. pDNA indicates the phosphorylated substrate DNA, while AppDNA indicates adenylated substrate by a DNA ligase. The substrate and the ligation product are labeled with arrows.

We decided to further investigate the ligation capabilities of rEhDNAligI in comparison with commercial T4 DNA ligase, to perform nick sealing of different DNA lesions located at the “bottom” or “template” strand of a synthetic DNA nick containing thymine dimers. Thymine dimers as a cyclobutane pyrimidine dimer (CPD) or a 6-4 photo product pose a strong block to replicate DNA polymerases and are only bypassed by specialized DNA polymerases. Thus, we tested three possible substrates with different base modification related to thymine dimers (Figure 3A). Our results indicate that rEhDNAligI was able to ligate a CPD when this lesion is located between the DNA junction, but not when it is located at the 3′-OH or 5′-PO_4_ (Figure 3B lane 2). In contrast T4 DNA ligase was also able to ligate a 6-4 photoproduct when the lesion was in the 3′-OH of the junction (Figure 3C lanes 2, 4). Neither of both DNA ligases were able to seal a thymine dimer, when the lesion was present at the 3′-OH side of the nick (Figures 3B,C lanes 3, 6).

### Survey of BER and NER gene encoding proteins in *E. histolytica* genome

*E. histolytica* encodes genes involved in the NER pathway, suggesting that this mechanism could be used by this parasite (López-Camarillo et al., 2009; Marchat et al., 2011). In our *in silico* analysis, we found orthologs for genes encoding the protein Cul4 and XPF and ERCC1 nucleases, which are involved in the release of DNA damage during the repair process (Table 2). Regarding BER pathway, we identified the monofunctional DNA glycosylase EhMUTY, EhAPEX, and EhFEN1 the strand resolving exonucleases (Table 2). Founding a homolog to the AP endonuclease APEX is relevant as previously it was reported the absence of a gene encoding an AP endonuclease in this parasite (López-Camarillo et al., 2009). All proteins from NER and BER pathways encoded in the *E. histolytica* genome described here, presented high percent of homology with proteins from *H. sapiens, S. cerevisiae*, and other eukaryotes (Table S2). Nevertheless, we found that some components from the BER pathway have high identity with proteins from bacteria suggesting that were acquired by lateral gene transfer (LGT).

**Table 2:** Proteins from the NER and BER pathways in *Entamoeba histolytica*.

**Putative function**	**Name**	**MW (kDa)**	**Annotation^&^**	RefSeq^*^	**Locus**
**NER**					
Damage recognition	EhR23-1^b,c^	34.7	RAD23 protein, putative	XP_649512	EHI_001400
	EhR23-2	40.7	UV excision repair protein RAD23, putative	XP_648488	EHI_106100
	EhDDB1^c^	12.3	DNA damage-binding protein, putative	XP_653855	EHI_194860
	EhCul4	81.5	Cullin family protein	XP_657526	EHI_148160
TFIIH opening of DNA	EhXPD^a,b^	89.9	DNA repair helicase, putative	XP_649489	EHI_132410
	Helicase^b^	88	DNA repair helicase, putative	XP_650832	EHI_197850
	EhXPB^a,b^	75.1	DNA repair helicase, putative	XP_649651	EHI_088430
	Helicase^b^	118.7	DNA repair helicase, putative	XP_651401	EHI_054240
	Helicase^b^	88.6	Helicase domain-containing protein	XP_649509	EHI_001430
	Ehp44^a,b^	42.5	TFIIH basal transcription factor complex subunit, putative	XP_655154	EHI_182880
	Ehp52^a^	57.2	Hypothetical protein	XP_656826	EHI_192400
	Ehp34^a,b^	29.7	Hypothetical protein, conserved	XP_650390	EHI_091540
	Ehp62^a^	44.4	Hypothetical protein	XP_651579	EHI_117670
	Ehp8^a^	7.6	Hypothetical protein	XP_648756	EHI_074550
ssDNA protection	EhRPA	66.6	Replication factor A protein 1, putative	XP_650233	EHI_062980
Release of damage DNA	EhXPG^b^	75.7	DNA-repair protein, putative	XP_651701	EHI_100400
	EhXPF	104.6	DNA repair endonuclease, putative	XP_657509	EHI_148310
	EhERCC1	27.8	DNA excision repair protein, putative	XP_652464	EHI_126160
**BER**					
**Damage recognition and base excision**
Monofunctional	EhUDG^c^	29.9	Uracil-DNA glycosylase, putative	XP_656294	EHI_178910
	EhMutY	35.4	A/G-specifi c adenine glycosylase, putative	XP_653060	EHI_010700
	EhAlkD^d^	39.8	Hypothetical protein, conserved	XP_653223	EHI_162220
Bifunctional	EhNth^b,c^	35.5	Endonuclease III, putative	XP_655025	EHI_083460
	EhNth-like^b,c^	27.5	Endonuclease III, putative	XP_654116	EHI_118790
AP site incision/excision	EhApex	37.4	Exodeoxyribonuclease III, putative	XP_650532	EHI_046670
	EhFen1	42.6	Flap nuclease, putative	XP_651270	EHI_099740
**BOTH PATHWAYS**					
DNA synthesis	EhRFC	35.6	Replication factor C subunit 4, putative	XP_651156	EHI_086540
	EhPCNA^f^	28.5	Proliferating cell nuclear antigen	XP_651510	EHI_128450
	EhPolA^g^	75.9	DNA-directed DNA polymerase, putative	XP_653960	EHI_073640
	EhPolδ ^g^	124.4	DNA polymerase delta catalytic subunit, putative	XP_654477	EHI_006690
DNA ligation	EhLigI^e^	78.1	DNA ligase I	XP_657595	EHI_111060

a *Bedez et al., 2013*,

b *Marchat et al., 2011*,

c *López-Camarillo et al., 2009*,

d *Alseth et al., 2006*,

e *Cardona-Felix et al., 2010*,

f *Cardona-Felix et al., 2011*,

g *Pastor-Palacios et al., 2010*.

* *NCBI reference sequence database*.

& *According to AmoebaDB*.

### DNA damage induces expression changes of NER and BER genes

**Figure 4:**
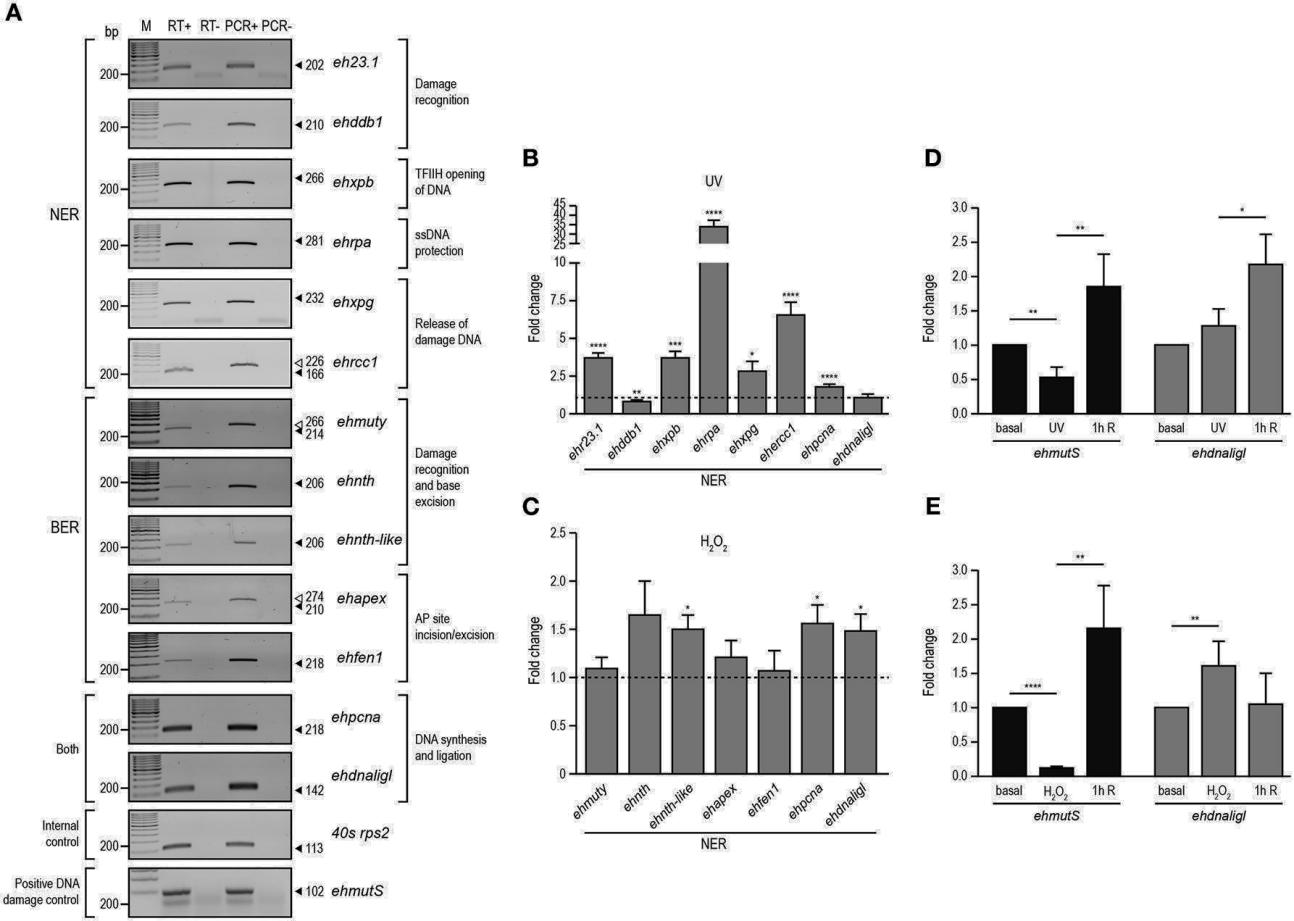
Expression of NER and BER genes in *E. histolytica*. RT-PCR analysis of NER and BER genes in basal conditions. **(A)** Expression of NER and BER genes using RNA isolated from trophozoites in basal conditions by semi-quantitative RT-PCR experiments (RT+). RT-: PCR assays using RNA without reverse transcriptase. PCR+: PCR assays using genomic DNA. PCR-: PCR experiments performed without genomic DNA. Arrowheads: expected size of the amplicons. qRT-PCR analysis of the NER **(B)** and BER **(C)** genes using RNA isolated from trophozoites treated with UV (150 J/m^2^) or H_2_O_2_ (2.5 mM for 1 h), respectively, compared with RNA obtained from trophozoites cultured in basal condition. **(D,E)** qRT-PCR analysis of *ehmutS* (positive DNA damage control) and *ehdnaligI* genes during basal, immediately treated and 1 h recovery from UV **(D)** or H_2_O_2_ **(E)** treatments. Fold change was determined using *40s rps2* as internal control gene. Data represent media ± standard error from three independent experiments. *p < 0.05, **p < 0.01, ***p < 0.001, ****p < 0.0001.

In *E. histolytica*, UV and H_2_O_2_ treatments have been used to produce oxidative stress, generating changes in gene expression (Vicente et al., 2009; Weber et al., 2009; Pearson et al., 2013). In order to investigate if BER and NER genes were regulated upon these treatments, we irradiated trophozoites with UV (150 J/m^2^) or incubated them with H_2_O_2_ (2.5 mM for 1 h at non-lethal conditions, as previously described (López-Casamichana et al., 2008; Shahi et al., 2016). Data showed that the viability of the trophozoites was 85% immediately after treatments, similar to those previously reported. However, between 1 and 2 h of recovery after treatments, we observed a decrease in the number of trophozoites (>50% of the viability) and eventually the viability was recovered after 3 h (Figure S2), suggesting that treatments produce DNA lesions that could be repaired allowing trophozoites to continue divide and multiply. First, in order to determine the expression patterns of NER and BER genes in *E. histolytica*, we performed RT-PCR assays of representative genes from each step of both pathways, in basal conditions. In basal culture conditions, trophozoites expressed all of the genes selected from the bioinformatics analysis (Figure 4A), suggesting the presence of both active DNA repair pathways in this parasite. We included minus reverse transcriptase controls, demonstrating that no genomic contamination was present in amplified genes. Additionally, positive PCR controls using genomic DNA were added, showing correspondence between the length of the amplicons obtained from genomic DNA and cDNA. We also included, the non-template control which eliminates possible false-positive results. Then, we analyzed changes in expression of NER and BER genes immediately after UV and H_2_O_2_ treatments using qRT-PCR. Results indicated that most of the NER genes increased their expression after irradiation treatment; *ehr23.1, ehxpb, ehrpa, ehxpg, ehercc*, and *ehpcna* genes presented 3.7, 3.6, 34, 2.8, 6.5, and 1.8-fold change, respectively compared with their basal condition (Figure 4B). Regarding genes of the BER pathway, only *ehnth-like, ehpcna*, and *ehdnaligI* increased their expression upon H_2_O_2_ treatment exhibiting 1.5, 1.6, and 1.5-fold change, respectively compared with their basal condition (Figure 4C). Furthermore, upregulation of the NER and BER genes suggest the activation of the DNA repair pathways. As positive control, we included the *ehmutS* gene (NCBI sequence ID: XM_647442) which encode for the DNA mismatch repair protein EhMutS. This gene was selected as a positive control because its expression is increased in response to both treatments in this parasite. The *ehmutS* gene is upregulated 1.55-fold after 3 h of recovery from UV injury and 2-fold during 1 mM H_2_O_2_ treatment (Vicente et al., 2009; Weber et al., 2009). In our conditions this gene was also upregulated after 1 h of recovery from UV and 2.5 mM H_2_O_2_ treatments, 2.4 and 2.1-fold respectively (Figures 4D,E), showing a response to DNA damage. Interestingly, *ehdnaligI* was upregulated immediately after H_2_O_2_ treatment (Figure 4C), and in UV injury *ehdnaligI* exhibited 2.1-fold-change after 1 h recovery (Figure 4D). This data showed that DNA ligases participate at the final steps of DNA repair pathways, therefore is important evaluate its expression during recovery from DNA damage.

### EhDNAligI induction during recovery from UV and H_2_O_2_ treatments

**Figure 5:**
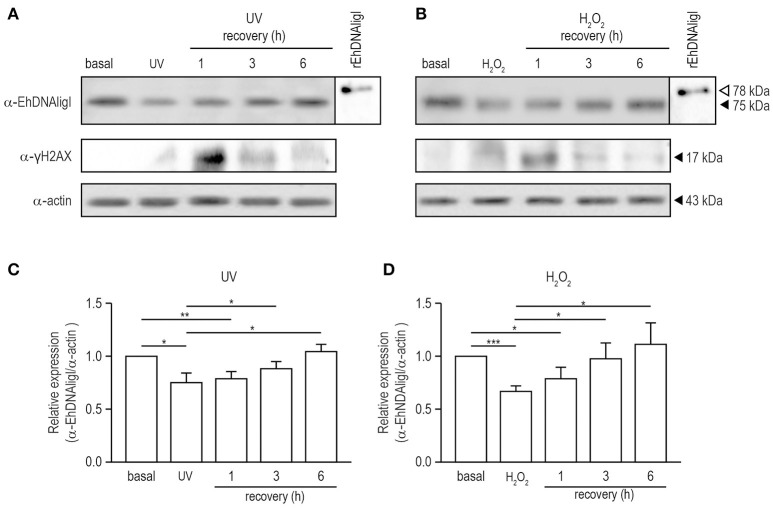
Expression of EhDNAligI in trophozoites recovered of DNA damage induced by UV and H_2_O_2_ treatments. **(A)** and **(B)** Total lysates from trophozoites in basal condition, immediately or recovered at different times from treatments with UV or H_2_O_2_ were immunodetected using the α-EhDNAligI antibody. γH2AX antibody was used as a control of DNA damage. α-actin antibody was used as loading control and rEhDNAligI was used as positive control. **(C,D)** Densitometry analysis of bands in **(A,B)**, respectively. Relative expression of EhDNAligI was normalized using actin and basal condition. Data represent media ± standard error from three independent experiments. *p < 0.05, **p < 0.01, ***p < 0.001.

To analyze the expression EhDNAligI protein in total extracts of trophozoites in basal condition and with both treatments at different times of recovery, we performed Western blot using a specific α-EhDNAligI antibody. This antibody detected a 75 kDa protein in trophozoite lysates (Figures 5A,B; Figure S1A) as previously described (Cardona-Felix et al., 2011). Immediately, after both treatments EhDNAligI level did not significantly raise, contrary to the expected we observed a decreased approximately 25–33%. An EhDNAligI increase of 30 and 44% at 6 h recovery of UV and H_2_O_2_ insults, respectively, was noticed. This late response could indicate that there could be a more complex regulatory pathway that would require additional experiments to understand it. As positive control, we included the rEhDNAligI recombinant protein, where the α-EhDNAligI antibody recognized a 78 kDa band, corresponding to the predicted molecular weight plus the histidine tag. We also included the anti-human γ-H2AX antibody that detects serine-phosphorylated EhH2AX homologue as an indicator of DNA damage. We identified a 17 kDa band, which corresponds to the expected molecular weight of γ-EhH2AX histones in *E. histolytica* (López-Casamichana et al., 2008). Trophozoites exhibited low levels of γ-EhH2AX in basal condition and increased after 1 h of recovery from both treatments and then it was diminished, indicating recovery from DNA damage. As a loading control, the α-actin antibody was used, and the relative protein expression was calculated after normalizing the EhDNAligI levels over the actin control (Figures 5C,D). The observed increase of EhDNAligI in response to UV and H_2_O_2_ treatments could be important during DNA repair following DNA insults.

### EhDNAligI co-localizes in nuclear regions where 8-oxoG is formed in trophozoites exposed to UV and H_2_O_2_ treatments

**Figure 6:**
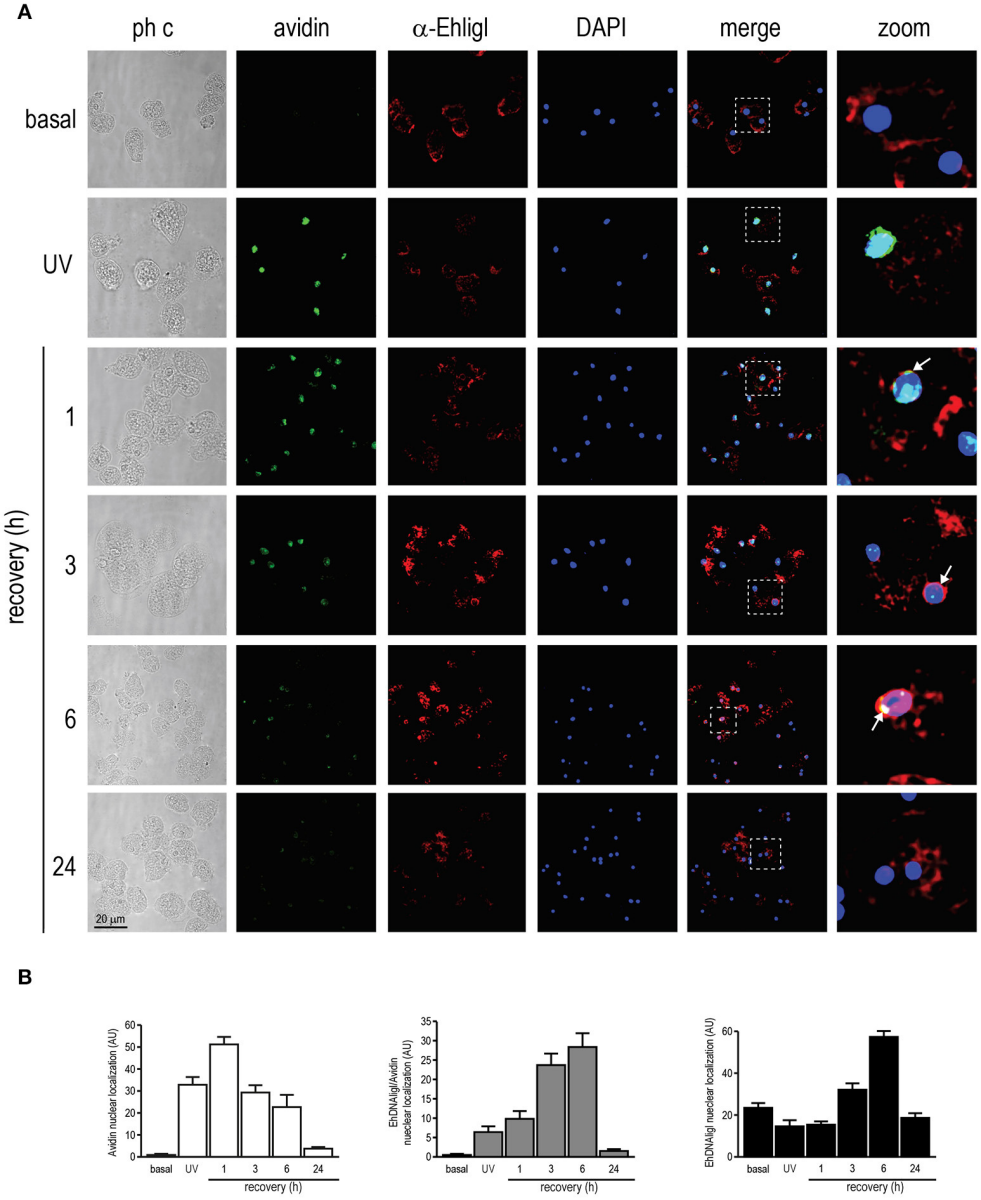
Co-localization of EhDNAligI with the 8-oxoG adduct after UV treatment in *E. histolytica* trophozoites. **(A)** Immunofluorescence assays of trophozoites in basal condition, immediately or recovered at different times after treatment with UV (150 J/m^2^). Trophozoites were incubated with avidin-FITC (Avidin) (green), and the α-EhDNAligI (α-EhligI) antibody (red). Nuclei were counterstained with DAPI (blue). Merge: overlapping of fluorescent channels. Confocal microscopy images show a *xy*-section. ph c: Phase-contrast. Arrows: co-localization of EhDNAligI and avidin-FITC. Squares were magnified in right panels. **(B)** Quantification of green (Avidin), red (EhDNAligI) and green/red fluorescence channels using DAPI staining as reference for the cell number.

**Figure 7:**
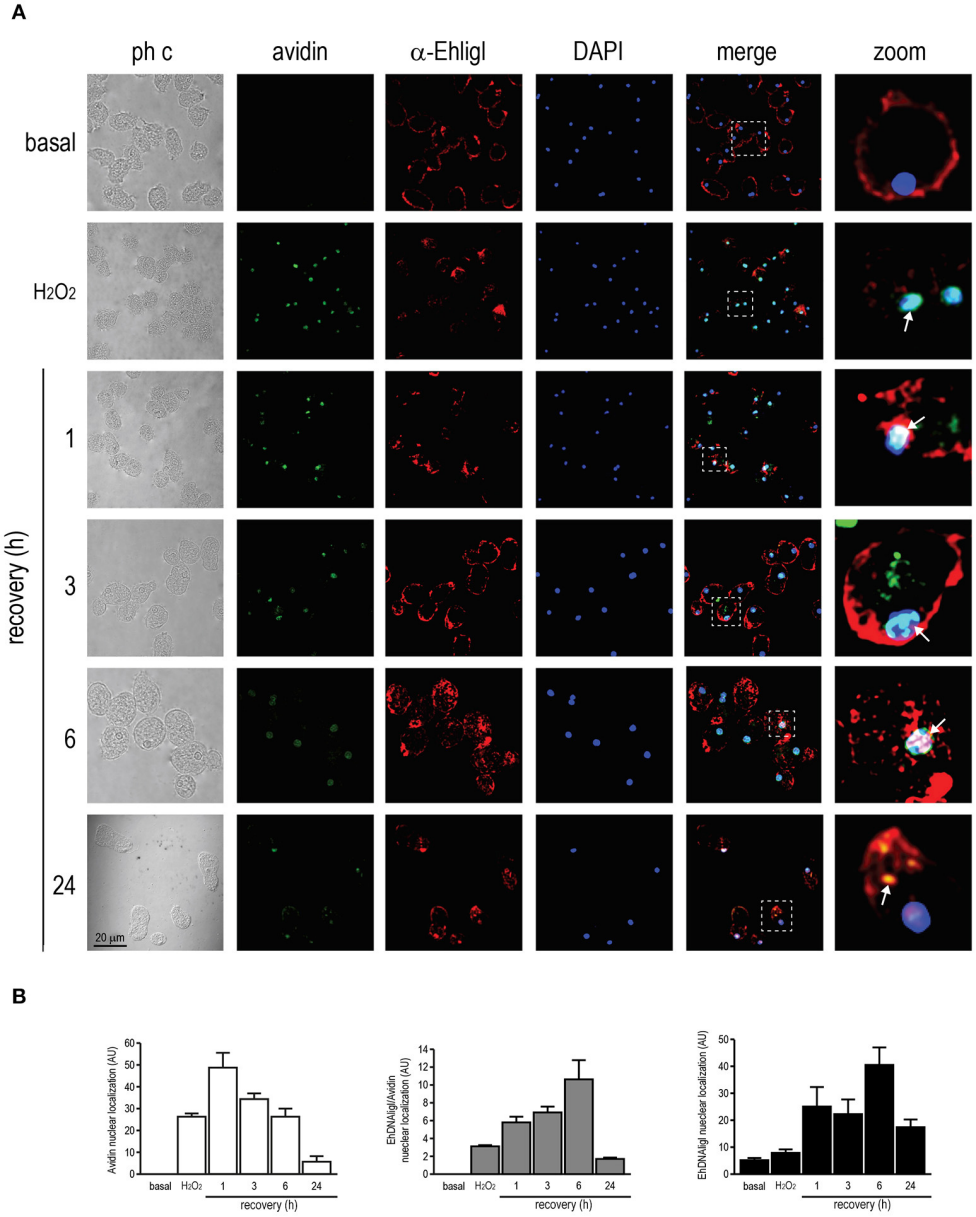
Co-localization of EhDNAligI with the 8-oxoG adduct after H_2_O_2_ treatment in *E. histolytica* trophozoites. **(A)** Immunofluorescence assays of trophozoites in basal condition, immediately or recovered at different times after treatment with H_2_O_2_ (2.5 mM for 1 h). Trophozoites were incubated with avidin-FITC (Avidin) (green), and the α-EhDNAligI (α-EhligI) antibody (red). Nuclei were counterstained with DAPI (blue). Merge: overlapping of fluorescent channels. Confocal microscopy images show a *xy*-section. ph c: Phase-contrast. Arrows: co-localization of EhDNAligI and avidin-FITC. Squares were magnified in right panels. **(B)** Quantification of green (Avidin), red (EhDNALigI) and green/red fluorescence channels using DAPI staining as reference for the cell number.

Here we have demonstrated that UV and H_2_O_2_ treatments produced changes in EhDNAligI expression. Nevertheless, the effects of these agents on the DNA integrity in this parasite have not been determined. Even though we are using two different treatments, it is well documented that UVC radiation may generate ROS, which consequently induce DNA damage (Wei et al., 1997; Zhang et al., 1997). Guanine is attacked preferentially upon oxidative DNA damage because it has the lowest oxidation potential of the four bases, resulting in the formation of 7,8-dihydro-8-oxoguanine (8-oxoguanine; 8-oxoG). This lesion is abundantly produced *in vivo* and it is used as a biomarker of oxidative DNA damage (Shigenaga et al., 1990; Shigenaga and Ames, 1991). In order to verify whether UV and H_2_O_2_ induced 8-oxoG formation in this parasite, we assessed 8-oxoG accumulation in *E. histolytica* trophozoites using avidin-conjugated FITC. Avidin binds with high specificity to 8-oxoG and it has been used to detect oxidative DNA damage in different cell types, including parasites (Struthers et al., 1998; Furtado et al., 2012). In basal conditions, no avidin-FITC staining was observed in the nucleus of trophozoites (Figures 6A, 7A). However, in trophozoites treated with UV or H_2_O_2_, the avidin-FITC signal was markedly observed at nuclei reaching the maximum 8-oxoG induction at 1 h recovey for both treatments, co-localizing with the DAPI staining (Figures 6B, 7B). These results suggest that both treatments induced the formation of an 8-oxoG adduct, indicative of oxidative DNA damage. To explore possible cellular localization changes of EhDNAligI during and after DNA damage, we carried out immunofluorescence assays in trophozoites during and recovered from the UV and H_2_O_2_ treatments, using the α-EhDNAligI antibody. In basal conditions, confocal images evidenced the presence of EhDNAligI at the cytoplasm, whereas only a weak signal in the nucleus of the trophozoites was observed. Interestingly, immediately after UV treatment, EhDNAligI was translocated to the nuclear periphery and co-localized with the 8-oxoG adduct detected by the avidin-FITC, in some trophozoites (Figure 6A). After 1 h recovery, EhDNAligI was located at the nuclear periphery in the majority of the trophozoites, co-localizing with the avidin-FITC signal. From 3 to 6 h, EhDNAligI increased its presence at the cytoplasm and nucleus, and some trophozoites showed a diffuse staining in the whole nucleus not only at the periphery. At this time, a decrease in the nuclear DNA avidin-FITC intensity was observed. After a 24 h recovery, only a few trophozoites showed EhDNAligI at the nuclear periphery and it was more abundant at cytoplasm (Figure 6A), probably because the 8-oxoG was eliminated or repaired. The avidin-FITC intensity and the nuclear localization of EhDNAligI were quantified in several fields. After 1 h treatment, trophozoites exhibited the higher DNA damage which correlates with the presence of the phosphorylated histone γ-H2AX. Meanwhile, the mayor nuclear presence of EhDNAligI occurred after 6 h of recovery (Figure 6B).

Immediately, after H_2_O_2_ treatment, EhDNAligI translocated from the cytoplasm to the nuclear periphery, in comparison with the basal condition. At 1 to 6 h of recovery, EhDNAligI and avidin-FITC signals co-localized at the nuclear periphery, however the avidin-FITC staining diminished during the recovery. After a recovery of 6 h, EhDNAligI diffusely localized into the nucleus and at 24 h its cytoplasmic and membrane localization indicated a basal localization pattern (Figure 7A). These nuclear localizations were corroborated by fluorescence quantification (Figure 7B). Data from UV and H_2_O_2_ treatments are consistent with the protein changes observed by Western blot analysis. Altogether these findings suggest that both treatments induce 8-oxoG formation, which reflects the DNA damage was produced and also suggests that the 8-oxoG is being repaired. The decrease in trophozoites viability after treatments, and its recovery from 3 to 12 h, also reinforce the notion that 8-oxoG is being repaired (Figure S2). Furthermore, the EhDNAligI co-localization with the DNA damage suggests that this enzyme could be involved in the DNA repair process.

### EhDNAligI co-localizes with EhPCNA during recovery from DNA damage by UV and H_2_O_2_ exposure

**Figure 8:**
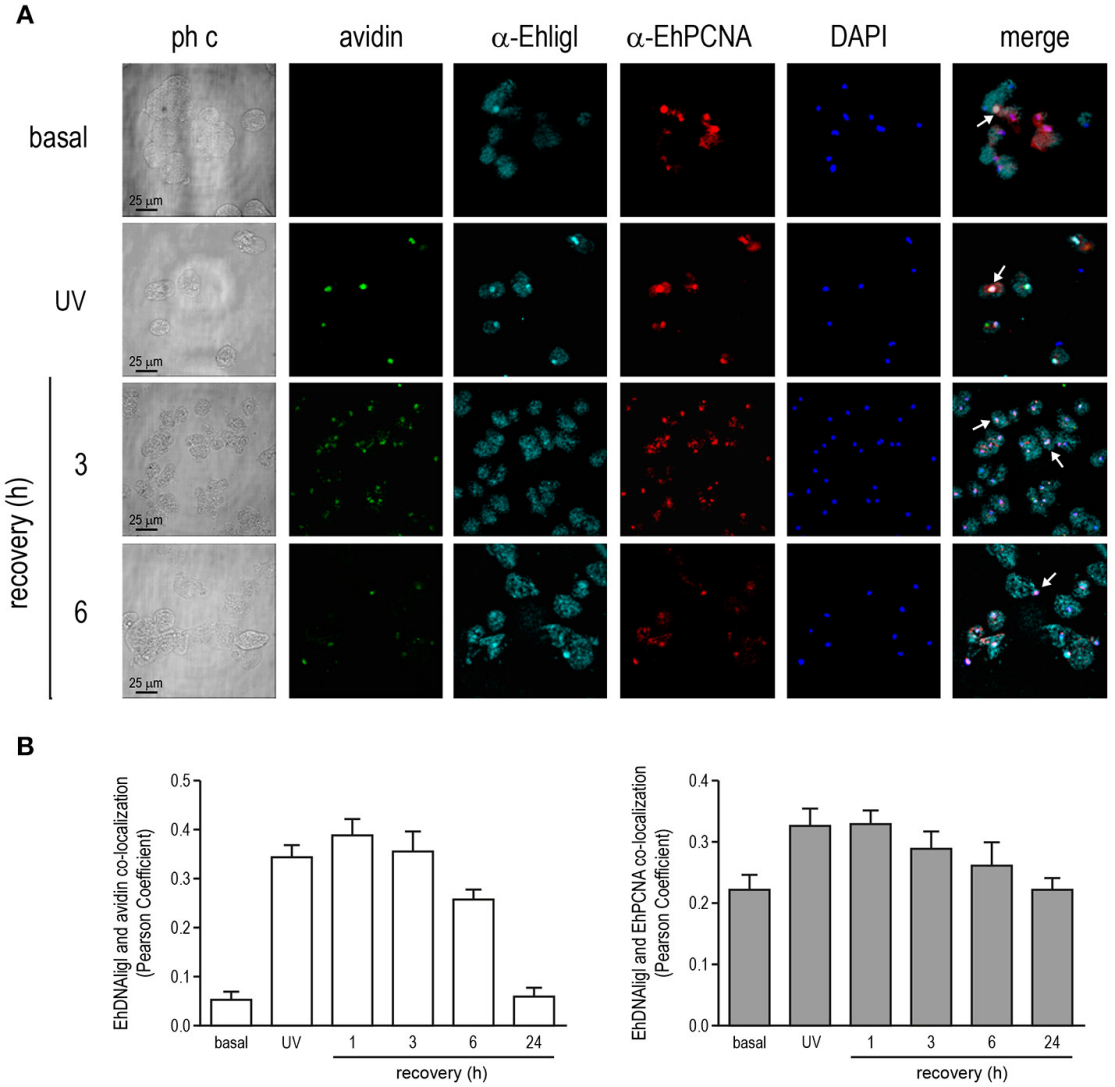
Co-localization of EhDNAligI with EhPCNA after UV treatment. Immunofluorescence assays of trophozoites in basal condition, immediately after treatment with **(A)** UV (150 J/m^2^) or at 3 and 6 h of recovery, using α-EhDNAligI (α-EhligI) and α-EhPCNA antibodies coupled to Alexa647 (cyan) and Alexa555 (red), respectively. 8-oxoG presence was detected with avidin-FITC (green). Nuclei were counterstained with DAPI (blue). Confocal microscopy images show a *xy*-section. ph c: Phase-contrast. Arrows: co-localization of EhDNAligI and EhPCNA. **(B)** Nuclear co-localization of avidin with EhDNAligI and EhDNAligI with EhPCNA was correlated by Pearson Coefficient.

**Figure 9:**
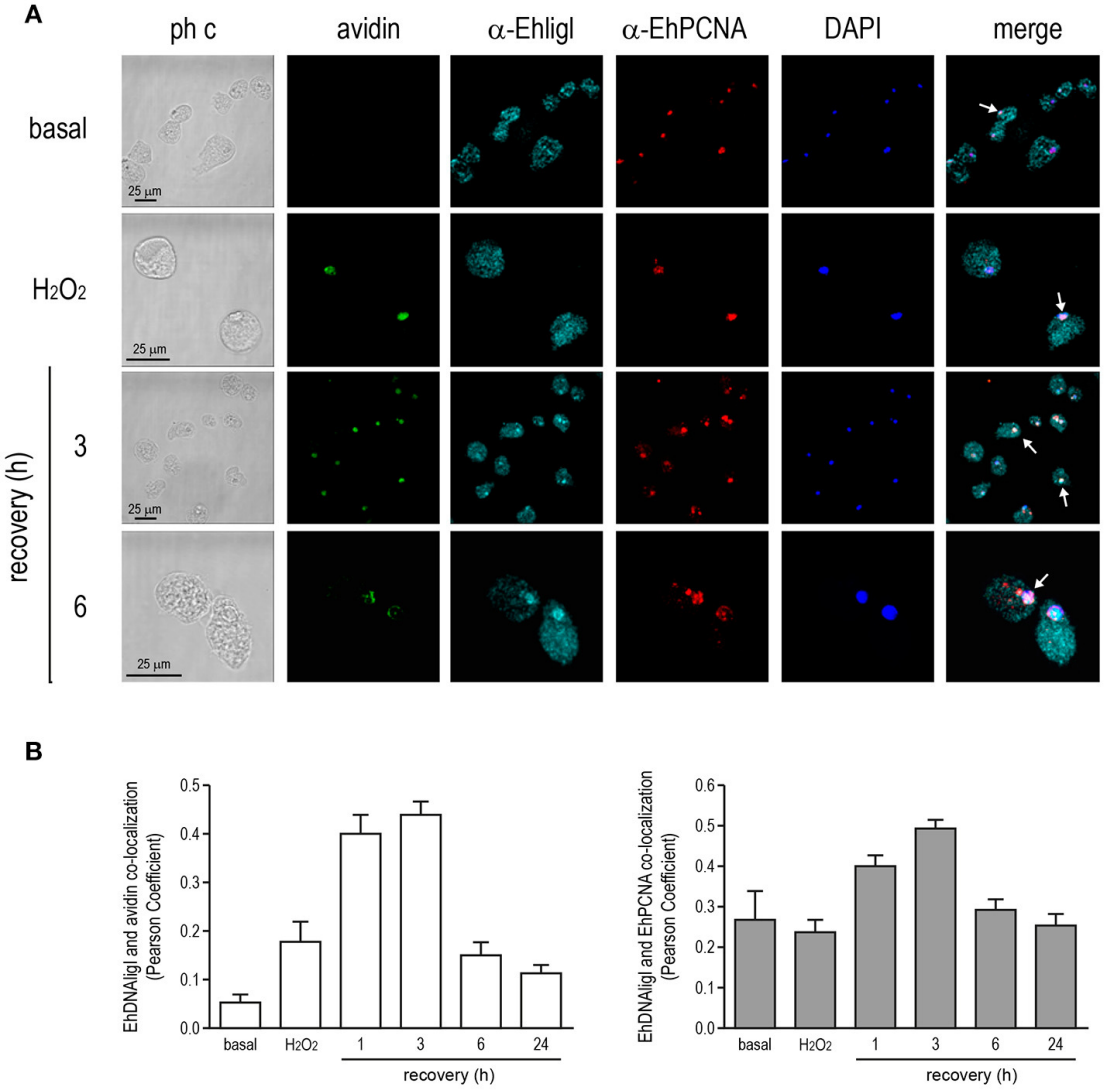
Co-localization of EhDNAligI with EhPCNA after H_2_O_2_ treatment. **(A)** Immunofluorescence assays of trophozoites in basal condition, immediately after H_2_O_2_ (2.5 mM for 1 h) or at 3 and 6 h of recovery, using α-EhDNAligI (α-EhligI) and α-EhPCNA antibodies coupled to Alexa647 (cyan) and Alexa555 (red), respectively. 8-oxoG presence was detected with avidin-FITC (green). Nuclei were counterstained with DAPI (blue). Confocal microscopy images show a *xy*-section. ph c: Phase-contrast. Arrows: co-localization of EhDNAligI and EhPCNA. **(B)** Nuclear co-localization of avidin with EhDNAligI and EhDNAligI with EhPCNA was correlated by Pearson Coefficient.

In several systems, DNA ligase I is recruited to DNA damaged sites by its interaction with PCNA (Mortusewicz et al., 2006). Moreover, EhDNAligI activity is enhanced by EhPCNA and these proteins present a functional interaction *in vitro* (Cardona-Felix et al., 2011; Trasviña-Arenas et al., 2017). Thus, we tested whether during recovery from UV and H_2_O_2_ treatments, EhDNAligI co-localizes with EhPCNA using an antibody generated against the rEhDNAligI recombinant protein (Cardona-Felix et al., 2010, 2011). By Western blot assays, this antibody recognized two bands in *E. histolytica* lysates, one of 28.5 kDa corresponding to the predicted molecular weight of EhPCNA, and another band of 75 kDa that may correspond to the trimeric ring assembly of EhPCNA (Figure S1B). The same bands were immunodetected when the rEhPCNA recombinant protein was used, demonstrating the specificity of the antibody (Figure S1B). In basal conditions, EhPCNA showed poor colocalization with EhDNAligI, where EhDNAligI presented a predominantly cytoplasmic stain while EhPCNA presented a more diffused pattern into the nuclei (Figures 8A, 9A). However, after DNA insults, EhDNAligI is relocated into the nucleus colocalizing with EhPCNA, with the higher colocalization rate immediately after UV treatment and 1 h recovery (Figure 8B) and 3 h recovery for H_2_O_2_ injury (Figure 9B). Additionally, EhDNAligI co-localized with avidin at the nuclei after insults, at the same times that it colocalized with EhPCNA. The EhDNAligI and EhPCNA co-localization inside the nuclei was mainly observed in the nuclear periphery and in some nuclear *foci*-like structure (Figures 8A, 9A). The co-localization of both proteins at nuclear *foci*-like structures during treatments recovery, suggest that EhPCNA recruits EhDNAligI toward DNA damage sites, indicating that EhPCNA is involved in targeting EhDNAlig to damaged sites but additional co-immunoprecipitation assays will necessary to strengthen the present results.

## Discussion

*E. histolytica* genome is subject to continuous DNA damaging agents. In order to survive and propagate, the parasite must cope with them. DNA repair pathways converge on the step of nick sealing, and in higher eukaryotes specialized DNA ligases are involved either in DNA repair or replication. DNA ligases are sensitive to mispairs on the 3′ side of the nick, but tolerant to mispairs on the 5′ or acceptor side (Sriskanda and Shuman, 1998a,b). The base pair geometry of 3′ base pairs is recognized by hydrogen bond interactions at the minor groove between DNA ligase and DNA duplex (Levin et al., 2004). Here, we found that rEhDNAligI only efficiently ligates the T:G mismatch at the 5′-phosphate strand, indicating a high discrimination level to non-canonical pairs. *Thermus thermophilus* and *E. coli*'s DNA ligase are also able to ligase a T:G mismatch (Chauleau and Shuman, 2016; Lohman et al., 2016). The wooble T:G pair creates less steric constrictions and is one of the most common mistakes by DNA polymerase β (Beard and Wilson, 1998) indicating that at the minor groove both enzymes are able to T:G pair as C:G, maybe by a water mediated interaction between the O_4_ of thymine and guanosine as the O_2_ of a cytosine. Thus, the biochemical data indicates that EhDNAligI is a high-fidelity DNA ligase. As this enzyme is the sole ligase present in the genome of *E. histolytica*, its high fidelity maybe a consequence of its dual role in DNA replication and DNA repair. Therefore, the EhDNAligI study is relevant as it could be a potential target for anti-parasitic agent.

Oxidative damage, alkylating agents, and UV light are the most prone agents to modify DNA and alter its coding capabilities (For reviews: Evans et al., 2004; Reardon and Sancar, 2004). Nick-sealing fidelity in DNA ligases relates to the extent to which the enzyme can ligate substrates containing mismatched bases on either side of the nick. The mutagenic potential of DNA lesions has been extensively studied in DNA polymerases, however few studies have been carried out with DNA ligases. Reactive oxygen species originates 8-oxoG and thymine glycol. DNA ligase III efficiently sealed a Tg lesion in the 3′ OH (Budworth and Dianov, 2003), however its ligation efficiency was not tested at the 5′ OH. EhDNAligI and T4 DNA ligase efficiently ligate a thymine glycol at a 3′OH, however they are inefficient in sealing a nick at the 5′OH. Crystal structures of thymine glycol with DNA polymerases indicate that the methyl group of thymine glycol affects catalysis by altering the orientation of the 3′ OH (Aller et al., 2007). Human DNA ligase is able to ligate a dATP or dCTP opposite 8-oxoG at the 3′ OH; however, dATP is ligated more efficiently (Hashimoto et al., 2004). This contrasts with rEhDNAligI, that discriminates a dCTP-8-oxoG pair. This discrimination is reminiscent to the one observed by family A of DNA polymerases that are more efficient in extending an 8-oxoG:dATP mismatch than an 8-oxoG:dCTP base pair (Brieba et al., 2004; Hsu et al., 2004). EhDNAlig I is unable to ligate an abasic site 3′-OH of the nick, however T4 DNA ligase moderately ligates an AP site having dATP, but not dCTP, opposite the lesion. EhDNAlig I efficiently ligates an abasic site 5′-PO4 to the lesion only when dATP is complementary to the lesion. In contrast, T4 DNA ligase efficiently ligates an abasic site 5′-PO4 to the nick opposite a purine and a pyrimidine. This discrimination could be important for repairing abasic sites in the *Entamoeba* genome, as abasic sites are highly mutagenic and require a rapid and efficient repair. CPD and 6-4 photoproducts are not efficiently ligated by EhDNAligI. EhDNAligI was able to ligate a CPD when this lesion is located between the DNA junction, but not when it is at the 3′-OH or 5′-PO4, this correlates with the lack of binding for EhDNAligI observed by EMSA. In contrast, weak binding was observed in the 6-4 photoproduct when the lesion is present at the 3′-OH or 5′-PO4, indicating that thymine dimers distort the DNA helix and the catalytic step (Figure S3). Eukaryotic and archaeal DNA ligase contain an extra N-terminal domain dubbed DNA binding domain that considered essential to detect a properly organized substrate. The lack of a DBD in a T4 ligase may be involved in its low fidelity and its ability to use damaged bases as substrates (Zhao et al., 2007).

Different DNA repair systems are involved in the preservation of the genome by correcting DNA lesions originated by damaging agents, such as UV light and H_2_O_2_. DNA ligases are involved in BER and NER, DNA double-strand breaks, DNA non-homologous end-joining and homologous recombination pathways. In this work, we focused in BER and NER repair pathways, which can be induced by H_2_O_2_ and UV light treatments, respectively. Even though different genes involved in NER and BER pathways have been identified in the *E. histolytica* genome (Alseth et al., 2006; López-Camarillo et al., 2009; Cardona-Felix et al., 2010; Pastor-Palacios et al., 2012; Bedez et al., 2013), in this study we extended these findings including new genes that we identified in our survey. The *E. histolytica* genome encodes for proteins involved in the DNA damage recognition, as Rad23 and DDB1, which could be involved in UV excision repair. The gene encoding for Cul4 is also present in the *E. histolytica* genome; this protein is part of the core involved in DNA damage response. The nine subunits of the TFIIH complex and the nucleases XPF and ERCC1 are also encoded by the *Entamoeba* genome. In the case of genes encoding proteins for the BER pathway, it genome contains putative monofunctional (UDG and AlkD) and bifunctional (NTH) glycosilases (Loftus et al., 2005). Our survey revealed genes encoding for the MutY glycosilase and strand resolving exonucleases as Apex and Fen1. Interestingly, EhUDG and EhMutY glycosylases presented high identity with bacterial proteins, suggesting that the horizontal gene transfer had a role in their acquisitions (Table S2). Functional studies of EhMutY revealed that this enzyme, like its bacterial counterparts, is functional, even it lacks an iron-sulfur cluster (Trasviña-Arenas et al., 2016). Apparently, the genome of *E. histolytica* does not encode for proteins required in short-patch BER, as DNA polymerase β, XRCC1 and DNA ligase III; thus, we speculate that this parasite uses the long-patch BER to cope with the oxidative stress produced in the colonic tissue or during the amebic liver abscess. In both NER and BER pathways, DNA gaps are filled using the information available on the complementary strand by a DNA polymerase, that in *E. histolytica* could be a DNA polymerase with high amino acid identity to bacterial DNA polymerase I (Pastor-Palacios et al., 2010). Eukaryotes express three DNA ligases, dubbed I, III and IV. DNA ligase I is the main nuclear ligase, DNA ligase III is involved in DNA repair at the mitochondria and nucleus, and DNA ligase IV is involved in non-homologous end-joining (NHEJ) (Nash et al., 1997; Ellenberger and Tomkinson, 2008) and DNA ligase I can be substituted for DNA ligase IV in NHEJ (Lu et al., 2016). Because *Entamoeba* does not contain associated organelles with DNA like apicoplast or mitochondria is plausible that EhDNAligI is involved in all the nick-sealing reactions during DNA replication or repair of this parasite. Genome reduction is observed in protozoan parasites, for instance *E. histolytica* and *Plasmodium falciparum* code for half and one quarter of the proteins reported for *H. sapiens*. In these protozoan parasites only one DNA ligase with similitude to DNA ligase I is reported (Buguliskis et al., 2007; Cardona-Felix et al., 2010). The lack of other ATP-dependent DNA ligases encoding genes involved in the DNA repair in the *E. histolytica* genome, suggests that EhDNAligI participates in BER, NER, and Okazaki fragment end-joining. But we cannot exclude the presence of noncanonical RNA ligases of the RtcB family that could directly ligate DNA 3′-phosphate with DNA 5′-OH ends (Das et al., 2013). Interestingly, *E. histolytica* genome encodes two proteins related the RctB family (NCBI sequence ID: XP_648794.1 and XP_651200.1). These proteins are annotated as hypothetical proteins and until now its RNA ligation function has not been characterized in this parasite. Remarkably, the genome of *Naegleria gruberi*, an amoeboflagellate, encodes for 13 canonical RNA ligases (seven from the Rnl1 family, five from the Rnl2 family, and one Rnl5 enzyme) and two noncanonical RNA ligases of the RtcB family. This plethora of RNA repair enzymes in this organism reflects a functional specialization of the ligase families for different types of DNA/RNA damages (Unciuleac and Shuman, 2015). In *E. histolytica* genome we didn't find encoded canonical RNA ligases using bacterial or *Trypanosome* RNA-editing ligases as a bait, leaving only the RNA ligases capable of binding DNA fragments.

In *E. histolytica*, few DNA binding proteins like EhDNAligI, EhPCNA and EhMuyY have been characterized, however little is known about how these proteins interact during DNA repair [10, 12]. Here, we shown that representative genes of BER and NER pathways are expressed in trophozoites, under basal culture conditions. In order to evaluate expression changes of NER and BER genes during different injuries, we selected UV and H_2_O_2_ treatments to produce DNA damage. Basal expression of NER genes was compared against trophozoites treated with UV irradiation. This treatment caused upregulation of most NER genes, but no changes in *ehdnaligI* transcript were observed. Nevertheless, during 1 h recovery *ehdnaligI* was upregulated. Regarding the 2.5 mM H_2_O_2_ treatment, we found upregulation of the *ehnthlike, ehpcna*, and *ehdnaligI* genes. An explanation for this difference could due to UV treatment involved a short time period, in contrast to 2.5 mM H_2_O_2_ injury which comprise 1 h incubation, suggesting that the induction of repair mechanisms was differentially activated. In another study, trophozoites treated for 1 h with 1 mM H_2_O_2_, 286 genes belonging to the detoxification systems were modulated; however, not significant changes in e*hdnaligI* or BER genes were observed (Vicente et al., 2009; Pearson et al., 2013). Even though, 1 mM of H_2_O_2_ is a physiologically concentration present in the gastrointestinal lumen (Mayol et al., 2006), we do not know if during the oxidative stress in colon or hepatic abscess, the H_2_O_2_ concentration is elevated and it produces expression changes in BER genes in response to DNA damage. Furthermore, we cannot discard modifications of BER genes or proteins or their recruitment to the DNA damage sites, during different DNA recovery treatments. Our findings suggest the presence and participation of NER and BER pathways during DNA damage. We included as a positive DNA damage control, the *ehmutS* gene, because in this parasite is upregulated during both DNA insults, as it has been widely studied for *Escherichia coli* (Vicente et al., 2009; Weber et al., 2009). Prokaryotic MutS and eukaryotic homologs are involved in the DNA mismatch repair (MMR) and have a major role in the mismatched DNA recognition; however, MMR has been also implicated in the response to oxidative response (Khil and Camerini-Otero, 2002; Mazurek et al., 2002; Germann et al., 2012). The 8-oxoG recognition systems involved several overlapping pathways common in bacteria, yeast, and human cells (Mazurek et al., 2002), thus we considered that *ehmutS* gene is a suitable positive control for determining induction of DNA damages responses in *E. histolytica*.

The *ehdnaligI* transcript was slightly upregulated only immediately after peroxide treatment, however its protein levels decreased after peroxide and UV treatments and increased from 1 to 6 h, reinforcing the idea about its participation during DNA repair. Similar results were obtained for DNA ligase I in fibroblasts exposed to UV irradiation by Montecucco and coworkers, where DNA lig I expression decreases immediately after treatment and then increases reaching a maximum 24 h after damaging treatment (Montecucco et al., 1992). Also, free radicals produced by UV and H_2_O_2_ treatments caused oxidative modifications of proteins, leading to changes in their physical and chemical properties, including conformation, structure, solubility, susceptibility to proteolysis, and enzymatic activities (Davies, 2001, 2005). Recently, a large-scale oxidized proteins have been identified in oxidatively stressed trophozoites treated for 1 h with 2.5 mM H_2_O_2_ (Shahi et al., 2016). This could explain the EhDNAligI diminish observed during UV and H_2_O_2_ treatments, assuming that these injures generated physical and chemical changes in EhDNAligI. Then, at 3 and 6 h recovery from both treatments, it is possible that the *ehdnaligI* transcription be stimulated to increase the amount of protein observed.

In previous reports, *E. histolytica* trophozoites have been exposed to several H_2_O_2_ concentration and UV irradiation (López-Casamichana et al., 2008; Vicente et al., 2009); however, oxidative damage of DNA has not been demonstrated. In this work, we determined that immediately after 2.5 mM H_2_O_2_ and UV (150 J/m^2^) light treatments the formation of the 8-oxoG adduct was induced, as a result of the DNA damage. The DNA damage was evaluated by avidin-FITC, which is widely used to assess the 8-oxoG accumulation due to structural similarities between biotin and 8-oxoG and the high affinity of avidin for biotin (Struthers et al., 1998). This approach has been also used in other parasites as *T. cruzi* to detect the adduct induction during treatment with 200 μM of H_2_O_2_ (Furtado et al., 2012). On the other hand, cells treated with UV light provide an experimental system for depicting the biological consequences of DNA damage. It has been previously demonstrated that irradiation of trophozoites with 254 nm UV light at 150 J/m^2^, induced double-stranded DNA breaks (López-Casamichana et al., 2008); however, formation of neither DNA photoproducts or 8-oxoG has been not determined. It is also reported that the 8-oxoG adduct is induced in human cells exposed to different wavelengths throughout the UV spectrum, causing formation of ROS through the generation singlet oxygen, H_2_O_2_, superoxide and hydroxyl free radicals (Batista et al., 2009). The genotoxic effect of UV light has been mainly attributed to the induction these oxidative species (Heck et al., 2003). The 8-oxoG, and other forms of oxidative damage, may play an important role in the induction of the biological effects caused by UV light treatment. In this work, we found that in *E. histolytica* both treatments (UV and H_2_O_2_) produced the 8-oxoG adduct, possibly due to free radicals that were formed during treatments. Further analyses are needed to quantify the ROS species generated by these treatments.

In human cells, specific recruitment of BER and NER components to DNA damage sites, where 8-oxoG, is present has been reported (Melis et al., 2013). Then, it is possible that in *E. histolytica*, when DNA is damaged, repairing proteins such as NER and BER components could be recruited to the lesion sites. Here, we observed co-localization of EhDNAlig and 8-oxoG, suggesting that this enzyme is recruited to the DNA damage sites. These results agree with the reported in HeLa cells treated with laser micro-irradiated or 5mM H_2_O_2_, where BER components (MUTYH glycosylase and DNA polymerase λ) co-localized with this injury (van Loon and Hubscher, 2009). DNA ligase I is recruited to DNA repair sites by interaction with PCNA. The interaction between PCNA and DNA ligase I is not only critical for the subnuclear targeting of the ligase, but also for coordination of the molecular transactions that occur during lagging-strand synthesis (Mortusewicz et al., 2006). This interaction is mediated by a conserved peptide interacting motif (PIP box) present at the N-terminal region of EhDNAligI (Cardona-Felix et al., 2010). Additionally, it has been demonstrated that EhPCNA assembles as a homotrimer to interact with and stimulate EhDNAligI (Cardona-Felix et al., 2011). Our microscopy observations revealed co-localization of these proteins at nuclei in the same time that 8-oxoG lesion co-localized with EhDNAligI, suggesting a direct participation of EhDNAligI and EhPCNA during DNA repairing. However, elucidate whether they are participating in both BER and NER pathways remains to be analyzed. The biological importance of the 8-oxoG adduct formation is due to its propensity to mispair with adenine residues, leading to an increased frequency of spontaneous G:C to T:A transversions. This fact may predispose *E. histolytica* to evolve toward an AT rich genome. Although BER remains as the preferred pathway for repairing the 8-oxoG lesion, NER factors contribute to repair this lesion (Parlanti et al., 2012). Thus, the interaction of EhDNAligI and EhPCNA proteins could be an indicator of the possible activation of NER and BER pathways in *E. histolytica*, however co-localization assays of EhDNAligI with proteins involved in these routes, could clarify the ligase participation. The cytoplasmic abundance of this enzyme could indicate another possible role in nicking-closing reaction not only in nucleus but also in cytoplasm, in response to mutagenic effects.

Taken altogether our results showed that EhDNAligI is a high-fidelity DNA ligase in comparison to T4 DNA ligase. EhDNAligI also discriminated erroneous base pairs from opposite DNA lesions. EhDNAligI decreased after DNA insults but it recovered at 6 h and co-localized with EhPCNA, where DNA lesions remain. Additional studies are needed to better understand the role of EhDNAligI or other NER and BER components during DNA repair and recombination processes in this parasite.

## Author contributions

All authors contributed equally to design and conception of this work. CC-F, GP-P, CT-A, CD-Q, and LB conducted the biochemical experiments. EA-L, AB, EC-O, RC-G, GG-R, and DH-Á collected *E. histolytica* experimental data. HC performed the *in silico* analysis. EA-L, AB, EO, and LB contributed to experimental design, intellectual input, interpreting data, and in writing the manuscript.

### Conflict of interest statement

The authors declare that the research was conducted in the absence of any commercial or financial relationships that could be construed as a potential conflict of interest.

## Supplementary material

Figure S1 Immunoreactivity of the antibodies generated against rEhDNAligI and rEhPCNA proteins. Trophozoites extracts, rEhDNAligI **(A)** and rEhPCNA **(B)** were separated in SDS-PAGE gels. Gels were Coomassie stained or processed for Western blot analysis, using α-EhDNAligI **(A)** or α-EhPCNA **(B)** antibodies. TE: total cell extracts from *E. histolytica* trophozoites. Black arrowheads: molecular weight predicted size of the detected proteins, 28.5 and 75 kDa for EhPCNA and EDNAligI, respectively. Gray arrowhead: trimeric form of rEhPCNA. Numbers at the left: molecular weight of standards. ^*^EhDNAligI degraded products.

Figure S2 Viability of *E. histolytica* trophozoites exposed to UV and H_2_O_2_ treatments. Trophozoites treated with **(A)** UV (150 J/m^2^) or **(B)** H_2_O_2_ (2.5 mM for 1 h) were incubated in TYI-S-33 medium for different times and then stained with Trypan blue dye. The number of living trophozoites was counted in a counting chamber under a light microscope.

Figure S3 EhDNAligI is able to bind to DNA damaged substrates. EMSA experiments with radiolabeled DNA substrate containing lesions at the 3′-OH end or 5′-PO4 end in comparison to a canonical substrate using increasing concentrations of EhDNAlig I (26, 52, and 104 fmol) and 300 fmol of the indicates DNA substrates. Reaction mixtures were loaded onto a 6% native polyacrylamide gel and revealed by phosphorimagery. The position of the EhDNAligI-DNA substrate complexes and the free substrate are indicated by arrows. **(A)** EMSA experiments using an undamaged DNA substrate. Lane 1 shows the migration of radioactive probe in the absence of EhDNAligI. **(B)** EMSA experiments with a CPD the lesion at the 3′-OH (lanes 1–3) or 5′-PO4 end (lanes 4–6) and covering both positions (lanes 7–9). **(C)** EMSA experiments with a 6–4 PP DNA lesion, the lines are represented as in **(B)**. **(D)** EMSA experiment with thymine glycol 5R-6S isomer (lanes 1–6) or a 56-6R isomer (lanes 7–12). The damaged bases are located either at the 3′-OH (lanes 1–3 and 7–9) or at the 5′-PO4 end (lanes 4–6 and 10–12). **(E)** EMSA experiment with an abasic site. The nucleotide opposite to the abasic site is either a cytidine (lanes 1–6) or an adenine (lanes 7–12). The damaged base is located as in **(C)**. **(E)** EMSA harboring an 8-oxo guanosine adduct. The nucleotide opposite to the 8-oxo guanosine is either a cytidine (lanes 1–6) or an adenine (lanes 7–12).

Table S1 Paired primers used for assessing the transcript levels of the NER and BER genes in *E. histolytica*.

Table S2 Comparison of NER and BER proteins from *E. histolytica, H. sapiens, S. cerevisiae*, and other organisms.
