# Corrigendum: The sole DNA ligase in *Entamoeba histolytica* is a high-fidelity DNA ligase involved in DNA damage repair

**DOI:** 10.3389/fcimb.2022.1023564

**Published:** 2022-12-16

**Authors:** Elisa Azuara-Liceaga, Abigail Betanzos, Cesar S. Cardona-Felix, Elizabeth J. Castañeda-Ortiz, Helios Cárdenas, Rosa E. Cárdenas-Guerra, Guillermo Pastor-Palacios, Guillermina García-Rivera, David Hernández-Álvarez, Carlos H. Trasviña-Arenas, Corina Diaz-Quezada, Esther Orozco, Luis G. Brieba

**Affiliations:** ^1^ Posgrado en Ciencias Genómicas, Universidad Autónoma de la Ciudad de México, Mexico City, Mexico; ^2^ Consejo Nacional de Ciencia y Tecnología, Mexico City, Mexico; ^3^ Departamento de Infectómica y Patogénesis Molecular, Centro de Investigación y de Estudios Avanzados del Instituto Politécnico Nacional, Mexico City, Mexico; ^4^ Laboratorio Nacional de Genómica para la Biodiversidad, Centro de Investigación y de Estudios Avanzados, Irapuato, Mexico

**Keywords:** EhDNAligI, protozoan, DNA insults, ligation, repairing, 8-oxoG adduct, NER and BER pathways

**Error in Figure/Table**

In the published article, there was an error in **FIGURE 1B**. Assessment of rEhDNAligI fidelity on nicked double stranded DNA mismatches, as published. In **FIGURE 1B**, part of the figure that corresponds to lanes 10 to 18 (G:T mismatch) was accidentally duplicated in the part of the figure that corresponds to the A:T pair (lanes 19 to 26). The corrected **FIGURE 1B** and its caption appear below.

**Figure 1B:**
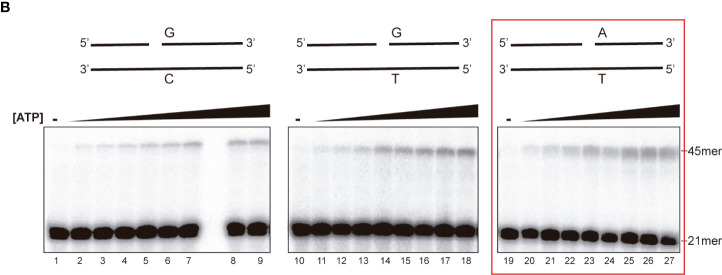
Steady-state kinetics of EhDNAligI on the G:T mismatch at different concentrations of ATP. Lanes 1–9 show EhDNAligI activity at different ATP concentrations in comparison to canonical G:C substrate, lanes 10–18 monitored ligase activity with a mismatched G:T substrate, lanes 19–27 indicate activity with the canonical A:T substrate. The 21mer substrate and the 45mer ligation products are marked with arrows.

The authors apologize for this error and state that this does not change the scientific conclusions of the article in any way. The original article has been updated.

## Publisher’s note

All claims expressed in this article are solely those of the authors and do not necessarily represent those of their affiliated organizations, or those of the publisher, the editors and the reviewers. Any product that may be evaluated in this article, or claim that may be made by its manufacturer, is not guaranteed or endorsed by the publisher.

