# Supplementary figures and images for: The Sole DNA Ligase in Entamoeba histolytica Is a High-Fidelity DNA Ligase Involved in DNA Damage Repair
Source: Front Cell Infect Microbiol. 2018 Jul 12;8:214. doi: 10.3389/fcimb.2018.00214 (PMC6052137; doi:10.3389/fcimb.2018.00214)

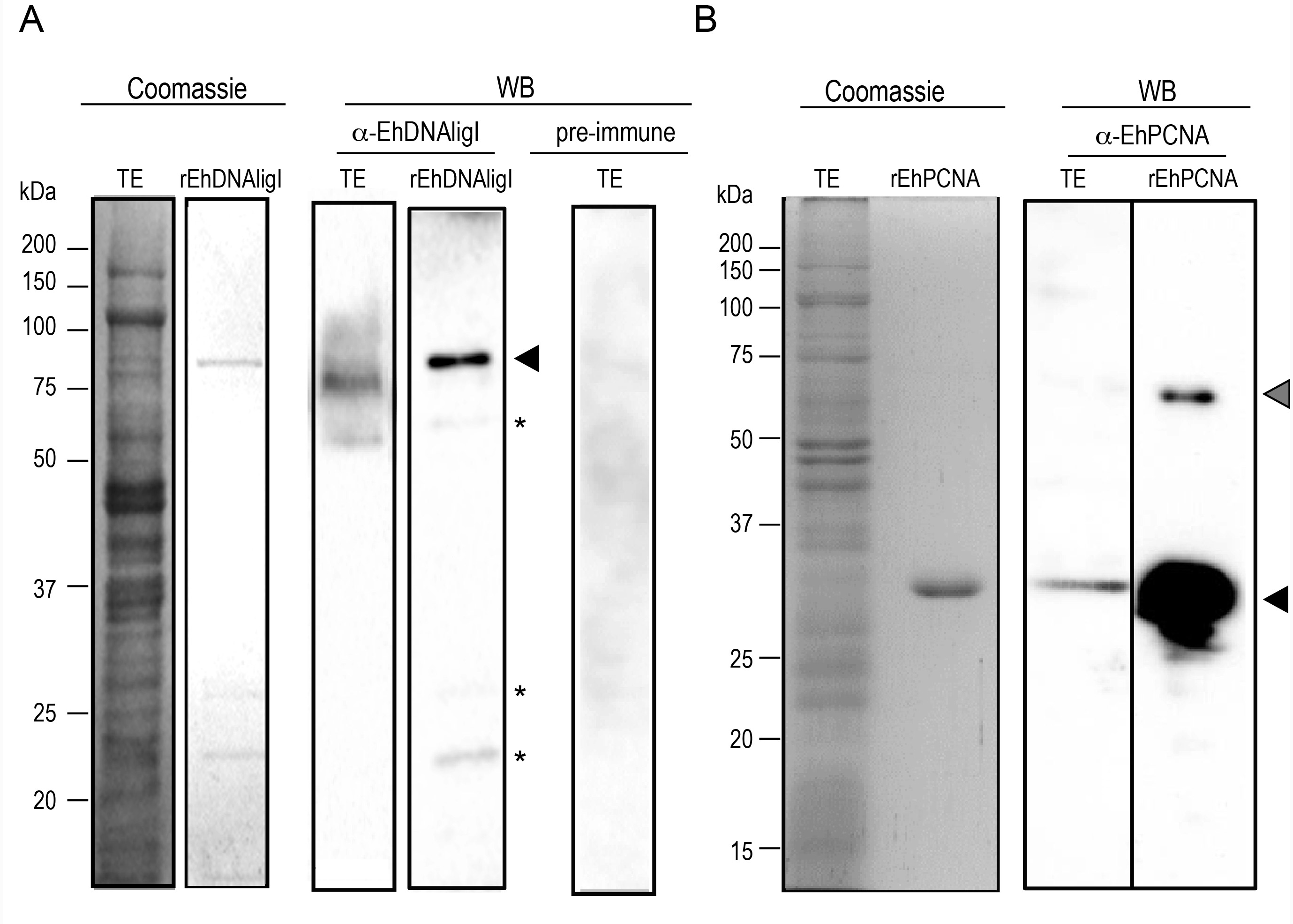

Supplement: Figure S1 — Immunoreactivity of the antibodies generated against rEhDNAligI and rEhPCNA proteins. Trophozoites extracts, rEhDNAligI (A) and rEhPCNA (B) were separated in SDS-PAGE gels. Gels were Coomassie stained or processed for Western blot analysis, using α-EhDNAligI (A) or α-EhPCNA (B) antibodies. TE: total cell extracts from E. histolytica trophozoites. Black arrowheads: molecular weight predicted size of the detected proteins, 28.5 and 75 kDa for EhPCNA and EDNAligI, respectively. Gray arrowhead: trimeric form of rEhPCNA. Numbers at the left: molecular weight of standards. *EhDNAligI degraded products. [file Image_1.JPEG]

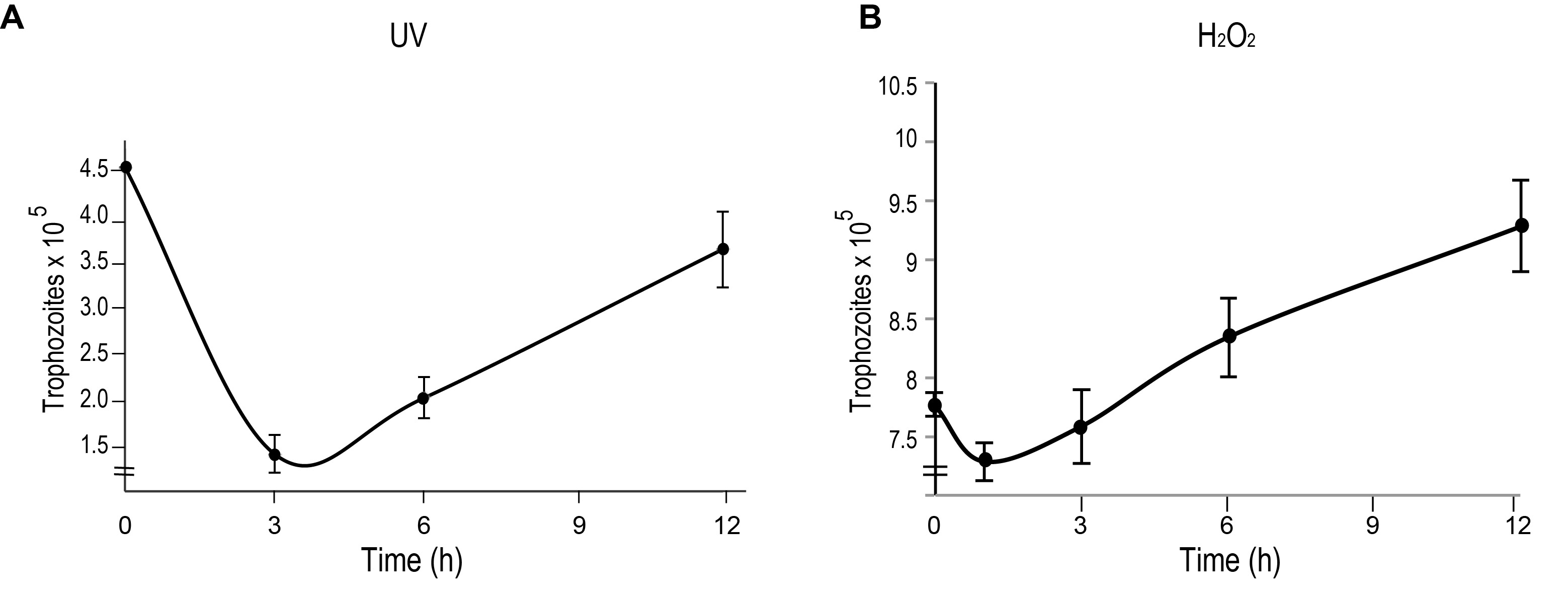

Supplement: Figure S2 — Viability of E. histolytica trophozoites exposed to UV and H2O2 treatments. Trophozoites treated with (A) UV (150 J/m2) or (B) H2O2 (2.5 mM for 1 h) were incubated in TYI-S-33 medium for different times and then stained with Trypan blue dye. The number of living trophozoites was counted in a counting chamber under a light microscope. [file Image_2.JPEG]

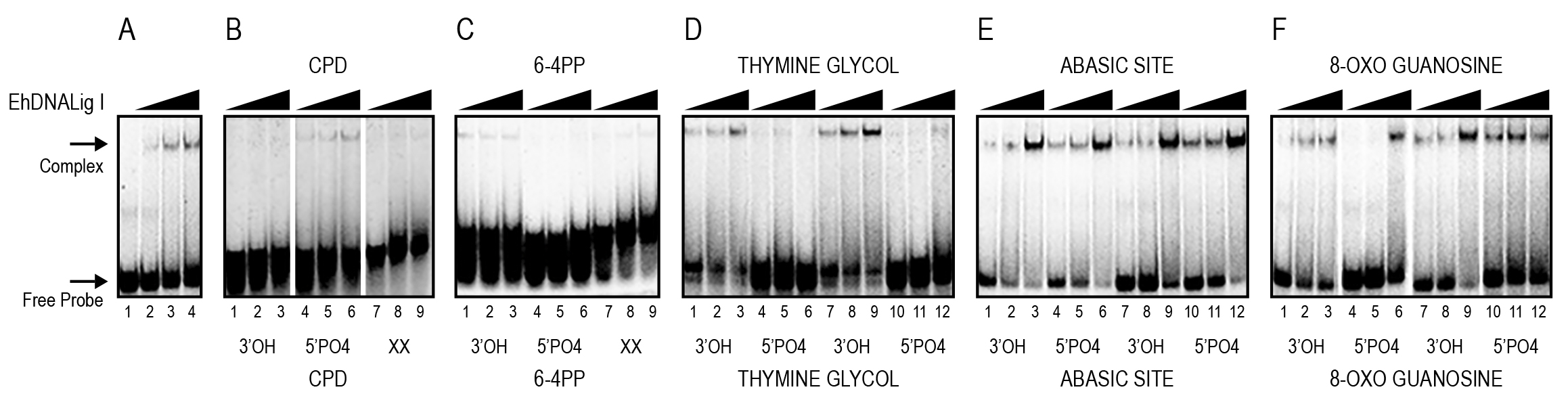

Supplement: Figure S3 — EhDNAligI is able to bind to DNA damaged substrates. EMSA experiments with radiolabeled DNA substrate containing lesions at the 3′-OH end or 5′-PO4 end in comparison to a canonical substrate using increasing concentrations of EhDNAlig I (26, 52, and 104 fmol) and 300 fmol of the indicates DNA substrates. Reaction mixtures were loaded onto a 6% native polyacrylamide gel and revealed by phosphorimagery. The position of the EhDNAligI-DNA substrate complexes and the free substrate are indicated by arrows. (A) EMSA experiments using an undamaged DNA substrate. Lane 1 shows the migration of radioactive probe in the absence of EhDNAligI. (B) EMSA experiments with a CPD the lesion at the 3′-OH (lanes 1–3) or 5′-PO4 end (lanes 4–6) and covering both positions (lanes 7–9). (C) EMSA experiments with a 6–4 PP DNA lesion, the lines are represented as in (B). (D) EMSA experiment with thymine glycol 5R-6S isomer (lanes 1–6) or a 56-6R isomer (lanes 7–12). The damaged bases are located either at the 3′-OH (lanes 1–3 and 7–9) or at the 5′-PO4 end (lanes 4–6 and 10–12). (E) EMSA experiment with an abasic site. The nucleotide opposite to the abasic site is either a cytidine (lanes 1–6) or an adenine (lanes 7–12). The damaged base is located as in (C). (E) EMSA harboring an 8-oxo guanosine adduct. The nucleotide opposite to the 8-oxo guanosine is either a cytidine (lanes 1–6) or an adenine (lanes 7–12). [file Image_3.JPEG]
