# Supplementary material for: The Sole DNA Ligase in Entamoeba histolytica Is a High-Fidelity DNA Ligase Involved in DNA Damage Repair
Source: Front Cell Infect Microbiol. 2018 Jul 12;8:214. doi: 10.3389/fcimb.2018.00214 (PMC6052137; doi:10.3389/fcimb.2018.00214)
Supplement: Table S1 — Paired primers used for assessing the transcript levels of the NER and BER genes in E. histolytica. [file Table_1.docx]

| **S1 Table. Paired primers used for assessing the transcript levels of the NER and BER genes in**  ***E. histolytica.*** | | | | | |
| --- | --- | --- | --- | --- | --- |
| **Gene** | **Ref Seq*** | **Use** | **Sequences of paired primers**  **(5´- 3´)** | **Amplicon size (bp)** | **TM** |
| **NER** | | | | | |
| *ehhr23.1* | XM_644420.1 | *RT-PCR* | F: GAGTGCTGTTCCTCCTCC  R: TCCCATTTCAACTAAATG | 202 | 53.91  47.39 |
|  |  | *qRT-PCR* | F: AAGAGTGCTGTTCCTCCTCC  R: ACTGGTTGTGTTGAAGGTTGG | 122 | 59.02  58.9 |
| *ehddb1* | XM_648763 | *RT-PCR* | F: GATGAACAAAGGGTTGTTG R: GAAATATCCCTCATTTCC | 210 | 52.19  47.49 |
|  |  | *qRT-PCR* | F: GGAATTAGTGGGGTTGTTATTGGG R: AGACAATACAACCCAACAACCC | 158 | 59.6  58.8 |
| *ehxpb* | XM_644397.1 | *RT-PCR* | F: TCGTGAAGATGATAGAATTCG  R: TAATTTTATCACCATGTGCC | 266 | 53.98  52.07 |
|  |  | *qRT-PCR* | F: CGAAGACAAGAGGCTCAACG  R: CCCTCTTTAGCAGGGTCTTCC | 253 | 58.94  59.79 |
| *ehrpa* | XM_645141.1 | *RT-PCR* | F: CTGATTACAAACAAAGTAATGGG  R: ATCTACTTCAACAGCATAGC | 281 | 54.25  48.13 |
|  |  | *qRT-PCR* | F: AGAAGGGAAGATCGCAGACG  R: TGAGATTGCAACAACGAGGG | 265 | 59.54  58.48 |
| *ehxpg* | XM_646609.2 | *RT-PCR* | F: CCTCTTTATCAGTCTATCCC R: TGGAAGTGTCTGGTGATGGG | 232 | 48.99  60.35 |
|  |  | *qRT-PCR* | F: CCATCTTCCTCCTCACTTCCC  R: AGAAGGAAGAGGGGAGAGAGG | 100 | 59.51  59.71 |
| *ehrcc1* | XM_647372.1 | *RT-PCR* | F: GATTAAAGGGATAAACTCGC  R: TGTTCCGACAATTGGGGAATG | 166 | 51.90  63.56 |
|  |  | *qRT-PCR* | F: GCGAGTTGAATATGATCCTGGC  R: GCAGCATCAGAATAACTTTGTGC | 282 | 59.52  59.14 |
| **BER** | | | | | |
| *Ehmuty* | XM_647968.1 | *RT-PCR* | F: TTACAACAAACACAAGTA  R: ATTTATCAACACTAGAAGG | 214 | 40.00  42.39 |
|  |  | *qRT-PCR* | F: TCAAGATATTGCAACAGTAGACGC  R:CCAGTTGCAACAAGAGCACC | 177 | 59.43  59.97 |
| *ehnth* | XM_649933.2 | *RT-PCR* | F: AAGTTGCATGAAACTCTT  R: GGTCCAACACCAGGAAGTG | 206 | 46.02  57.28 |
|  |  | *qRT-PCR* | F: ACACTTCCTGGTGTTGGACC  R: TCTGGGGTTGAACCATCTGC | 137 | 59.82  59.96 |
| *ehnth-like* | XM_649024.1 | *RT-PCR* | F: TTAAATGAGTTATTAGATGG  R: TGGTCAACAGTAATACCTTC | 206 | 43.31  48.47 |
|  |  | *qRT-PCR* | F: ACCTGGTATTGGTCCTAAGTTAGC  R: AGGACATTGGTCACATAAAGGG | 259 | 59.84  58.03 |
| *Ehapex* | XM_645440.2 | *RT-PCR* | F: CCTGATATTTTATGTGTTC  R: TTTCTCATATTCAACTGT | 210 | 43.45  40.71 |
|  |  | *qRT-PCR* | F: GTTGCAAGTTGGAATGCTGC  R: CCAGCATAACCTTTCTTAGCCG | 170 | 58.86  59.39 |
| *ehfen1* | XM_646178.1 | *RT-PCR* | F: GAAGGTACTTGTGCTGCATTAG  R: CATCCAAGTAAAATACAAAGG | 218 | 55.35  50.40 |
|  |  | *qRT-PCR* | F: ACCACCAGAAATGAAGGATGG  R: GCAGCACAAGTACCTTCTGC | 231 | 57.91  59.48 |
| **Both**  **Pathways** | | | | | |
| *ehpcna* | XM_646418.1 | *RT-PCR* | F: GCACTTGACAATGATTGTGG  R: GTAATTTTGAGGAATTCTGC | 200 | 55.89  50.19 |
|  |  | *qRT-PCR* | F: TCCAGAACTTCAATCTGACG  R: AGCACCTTTAGTAGTGAGAG | 142 | 52  51 |
| *ehdnaligI* | XM_652503.2 | *RT-PCR* | F: GATTATTTAAATGGATTAG  R: AGTCTTCAAATGTTCATAC | 259 | 40.13  42.52 |
|  |  | *qRT-PCR* | F: GATTAGCCGATACAGTTGAC  R: ATCTCCACTGTCTTCATCTC | 113 | 50.5  51.1 |
| **Positive DNA Damage control** | | | | | |
| *ehmutS* | XM_647442.1 | *qRT-PCR* | F: ATTCTATCCGTCCAACAACC  R: GAAGTTCAAACCCCTTTTGG | 104 | 55.1  54.9 |
| **Internal control** | | | | | |
| *40s rps2* | XM_643876.2 | *qRT-PCR* | F: ATTCGGAAATAGAAGAGGAGG  R: ACTAATCTT CCAAGCTTGGT | 105 | 57.5  54.3 |

^&^According to NCBI
