# Supplementary material for: The Sole DNA Ligase in Entamoeba histolytica Is a High-Fidelity DNA Ligase Involved in DNA Damage Repair
Source: Front Cell Infect Microbiol. 2018 Jul 12;8:214. doi: 10.3389/fcimb.2018.00214 (PMC6052137; doi:10.3389/fcimb.2018.00214)
Supplement: Table S2 — Comparison of NER and BER proteins from E. histolytica, H. sapiens, S. cerevisiae, and other organisms. [file Table_2.DOCX]

| **S2 Table. Comparison of *E. histolytica, H. sapiens, S. cerevisiae* and other organisms NER and BER proteins.** | | | | | | | | | | |
| --- | --- | --- | --- | --- | --- | --- | --- | --- | --- | --- |
| ***H. sapiens*** | | |  | ***S. cerevisiae*** | |  | **Other** | | | |
| **Name** | **RefSeq*** | **PID^&^** |  | **RefSeq*** | **PID** |  | **RefSeq*** | **PID** | **Species** |  |
| EhR23-1 | NP_005044 | **24.53** |  | NP_010877 | 20.5 |  | XP_001008308 | 22.22 | *Tetrahymena thermophila* | |
| EhR23-2 | NP_005044 | 19.13 |  | NP_010877 | 18.18 |  | XP_006690019 | **22.42** | *Candida tenuis* | |
| EhDDB1 | NP_001914 | 23.1 |  | NP_010587 | 13.35 |  | XP_008776760 | **25.39** | *Phoenix dactylifera* | |
| EhCul4 | NP_001008895 | **22.56** |  | NP_010150 | 18.36 |  | XP_012251214 | 22.27 | *Athalia rosae* | |
| EhXPD | NP_000391 | 28.55 |  | NP_011098 | 27.29 |  | XP_647302 | **28.82** | *Dictyostelium discoideum* | |
| EhXPB | NP_000113 | **30.71** |  | NP_012123 | 29.08 |  | XP_003662793 | 29.33 | *Myceliophthora thermophila* | |
| Ehp44 | NP_001506 | 30.95 |  | NP_013105 | 24.83 |  | XP_003737828 | **33.58** | *Metaseiulus occidentalis* | |
| Ehp52 | NP_001508 | 18.51 |  | NP_015203 | 16.95 |  | XP_001751645 | **19.61** | *Physcomitrella patens* | |
| Ehp34 | NP_001507 | 19.7 |  | NP_015381 | 16.42 |  | XP_003283083 | **25.08** | *Dictyostelium purpureum* | |
| Ehp62 | NP_001135779 | 11.39 |  | NP_010597 | 10.43 |  | XP_001641355 | 11.55 | *Nematostella vectensis* | |
| Ehp8 | NP_997001 | **16.86** |  | NP_076886 | 13.63 |  | NSA^#^ | - | *-* |  |
| EhRPA | NP_002936 | 19.94 |  | NP_009404 | 17.68 |  | XP_007554406 | **22.83** | *Poecilia formosa* | |
| EhXPG | NP_000114 | 12.8 |  | NP_011774 | **15.04** |  | XP_003065194 | 15.03 | *Coccidioides posadasii* | |
| EhXPF | NP_005227 | 19.3 |  | NP_015303 | 17.94 |  | XP_001812786 | **21.05** | *Tribolium castaneum* | |
| EhERCC1 | NP_001974 | 13.71 |  | NP_013614 | 11.18 |  | XP_004597998 | **15.8** | *Ochotona princeps* | |
| EhUDG | NP_550433 | 37.12 |  | NP_013691 | 31.47 |  | WP_018692098 | **43.98** | *Algicola sagamiensis* | |
| EhMutY | NP_001041636 | 17.16 |  | NP_009387 | 8.68 |  | WP_012502346 | **41.69** | *Chlorobaculum parvum* | |
| EhAlkD | NSA | - |  | NSA | - |  | XP_002668790 | **11.06** | *Naegleria gruberi* | |
| EhNth | NP_002519 | **29.62** |  | NP_009387 | 23.36 |  | XP_008891591 | 29.5 | *Phytophthora parasitica* | |
| EhNth-like | NP_002519 | 17.04 |  | NP_009387 | 16.39 |  | NP_228177 | **21.71** | *Thermotoga maritima* | |
| EhApex | XP_00526739 | 29.66 |  | NP_009534 | 6.89 |  | WP_016147086 | **34.78** | *Butyricicoccus pullicaecorum* | |
| EhFen1 | NP_004102 | 50.66 |  | NP_012809 | 43.71 |  | XP_005105611 | **51.98** | *Aplysia californica* | |
| EhRFC | NP_852136 | 34.18 |  | NP_014547 | 36.19 |  | XP_006454048 | **41.64** | *Agaricus bisporus* | |
| EhPCNA | NP_002583 | 33.08 |  | NP_009645 | 33.96 |  | NP_596504 | **38.11** | *Schizosaccharomyces* | |
| EhPolA | NP_861524 | 18.37 |  | NP_013643 | 10 |  | WP_026474298 | **21.48** | *Alkaliexus imshenetskii* | |
| EhPolδ | NP_002682 | 42.55 |  | NP_010181 | 42.16 |  | XP_003196775 | **46.7** | *Cryptococcus gattii* | |
| EhLigI | NP_001275993 | 28.45 |  | NP_010117 | 28.73 |  | XP_003283408 | **34.22** | *Dictyostelium purpureum* | |

*NCBI reference sequence database.

^&^Percentage identity of global alignment.

^#^No signicant alignment.

Bold letters indicate the highest PID value.
